# Community-Based Culturally Tailored Education Programs for Black Communities with Cardiovascular Disease, Diabetes, Hypertension, and Stroke: Systematic Review Findings

**DOI:** 10.1007/s40615-022-01474-5

**Published:** 2022-12-12

**Authors:** Hardeep Singh, Joseph Fulton, Sofia Mirzazada, Marianne Saragosa, Elizabeth M. Uleryk, Michelle L. A. Nelson

**Affiliations:** 1 Department of Occupational Science & Occupational Therapy, Temerty Faculty of Medicine, University of Toronto, 500 University Avenue Toronto, Toronto, ON M5G 1V7 Canada; 2 KITE, Toronto Rehabilitation Institute, University Health Network, 520 Sutherland Drive, Toronto, ON Canada; 3 Temerty Faculty of Medicine, Rehabilitation Science Institute, University of Toronto, 500 University Avenue Toronto, Toronto, ON M5G 1V7 Canada; 4 Institute of Health Policy, Management and Evaluation, University of Toronto, Toronto, ON Canada; 5 March of Dimes Canada, 10 Overlea Blvd, Toronto, ON Canada; 6 Bridgepoint Collaboratory for Research and Innovation, Lunenfeld-Tanenbaum Research Institute, Sinai Health System, 1 Bridgepoint Drive, Toronto, ON Canada; 7 E. M. Uleryk Consulting, Toronto, ON Canada

**Keywords:** Culturally tailored, Community-based, Systematic review, Health equity

## Abstract

**Background:**

Community-based culturally tailored education (CBCTE) programs for chronic diseases may reduce health disparities; however, a synthesis across chronic diseases is lacking. We explored (1) the characteristics and outcomes of CBCTE programs and (2) which strategies for culturally appropriate interventions have been used in CBCTE programs, and how they have been implemented.

**Methods:**

A systematic review was conducted by searching three databases to identify empirical full-text literature on CBCTE programs for Black communities with cardiovascular disease, hypertension, diabetes, or stroke. Studies were screened in duplicate, then data regarding study characteristics, participants, intervention, and outcomes were extracted and analyzed. Cultural tailoring strategies within programs were categorized using Kreuter and colleagues’ framework.

**Results:**

Of the 74 studies, most were conducted in the USA (97%) and delivered in one site (53%; e.g., church/home). CBCTE programs targeted diabetes (65%), hypertension (30%), diabetes and hypertension (1%), cardiovascular disease (3%), and stroke (1%). Reported program benefits included physiological, medication-related, physical activity, and literacy. Cultural tailoring strategies included peripheral (targeted Black communities), constituent-involving (e.g., community informed), evidential (e.g., integrated community resources), linguistic (e.g., delivered in community’s dialect/accent), and sociocultural (e.g., integrated community members’ religious practices).

**Conclusions:**

CBCTE programs may have beneficial outcomes, but a small sample size limited several. The strategies identified can be adopted by programs seeking to culturally tailor. Future interventions should clearly describe community members’ roles/involvement and deliver programs in multiple locations to broaden reach.

**Trial Registration:**

PROSPERO CRD42021245772.

**Supplementary Information:**

The online version contains supplementary material available at 10.1007/s40615-022-01474-5.

## Introduction

Chronic diseases are among the top causes of death and disability in many countries worldwide, including Canada and the USA [1–3]. According to the Center for Disease Control, 60% of adults in the USA have at least one chronic disease, and 40% have two or more [4]. A similar trend exists in Canada, with approximately 33% of adults living with a chronic disease [2]. Chronic diseases have a high economic burden, requiring significant annual healthcare and indirect costs (e.g., income and productivity loss) [5–7]. Chronic diseases are a concern as the burden is growing globally due to the aging population [8, 9].

Chronic diseases disproportionately impact certain ethnically marginalized groups and communities [10, 11], leading to significant health, economic, and psychosocial consequences [12, 13]. Ethnically marginalized groups in Canada and the USA experience a high prevalence of chronic diseases [1–7, 14, 10, 11, 15–17]. In particular, Black communities experience a higher risk and burden of chronic disease, including cardiovascular disease, hypertension, diabetes, and stroke, at a younger age than other ethnic groups [15–21]. In addition, they also exhibit a higher risk of chronic disease mortality and disability and experience chronic diseases younger than White adults [22–27].

Biological factors (e.g., genetic risk factors) only explain some of the disparities in chronic diseases, as multiple indirect and systemic factors also contribute to health disparities [21, 25, 28, 29]. Health inequities are partly due to economic, environmental, behavioral, and social factors [21]. For instance, statistics reveal that Black adults are less likely to visit a healthcare provider and spend less time engaging in self-care activities (e.g., blood glucose testing) than White adults due to multiple barriers that cause difficulty in managing chronic conditions [22, 30, 31]. These barriers can include the cost of accessing health and social services, a lack of access to physical activity facilities within their communities and difficulties acquiring fresh/healthy food [32, 33]. Moreover, dietary habits, stress, and negative interactions with the health system (e.g., discrimination) are also potential causes of poorer disease management behaviors [10, 21, 34]. When accessing health services, literature shows that Black adults receive lower quality care than other ethnic groups, and they may experience poor quality of communication and relationships with providers and less satisfaction with health services [5, 35–37]. Traditional health services may not adequately meet Black communities’ needs, as evidenced by multiple calls and initiatives to explore culturally tailored health service models [20, 38–42].

Culturally tailored programs (sometimes used interchangeably with culturally appropriate and cultural targeting) are defined in research as “the adaptation of the study design, materials and other components of the intervention to reflect cultural needs and preferences at the population level” [35, 43]. Culturally tailored programs have been recommended as a service delivery model to reduce health inequities [20, 22, 35, 43]. Prior reviews have examined culturally tailored programs for various communities, including Chinese American [44], Korean American [45], and other culturally diverse communities [43, 46, 47]. Culturally tailored programs can increase disease knowledge, health system access, clinical outcomes, and service satisfaction [35, 43, 44, 47, 48]. However, one review noted that culturally matched and linguistically appropriate education for immigrant Korean American community members might not be sufficient for chronic disease management, and culturally sensitive support should be provided [45]. It is unclear whether prior findings extend to community-based culturally tailored education (CBCTE) programs for Black communities. For purposes of this review, use of the term Black adults/communities reflects diverse communities who have African ancestry (e.g., African American, African Canadian, African-Caribbean, etc.); however, we acknowledge that the preferred terminology/language to describe one’s ethnic and cultural identity (e.g., African American/Black communities and ethnically marginalized communities) may differ among individuals and overtime.

While numerous CBCTE programs have emerged for Black adults with chronic diseases, such as cardiovascular disease [49], hypertension [50], diabetes [51–53], and stroke [54], cultural tailoring strategies have not yet been compared across chronic disease programs [55]. Two previous reviews examined culturally tailored diabetes interventions for Black adults [31, 56] and have added valuable insights into diabetes-specific culturally tailored programs for these communities. However, these findings may not be transferrable to other chronic conditions. A synthesis of programs targeting multiple common chronic conditions (i.e., cardiovascular disease, hypertension, diabetes, and stroke) is warranted to address the knowledge gap in the design and structure of “culturally tailoring” within these programs. This knowledge is necessary to guide the advancement of CBCTE programs to manage the growing burden of these chronic diseases facing Black communities [15–21].

### Objectives

The current review will address the following questions, as outlined in our protocol [55]:

1. What are the program characteristics and outcomes of CBCTE programs designed to improve health outcomes in Black adults with cardiovascular disease, hypertension, diabetes, or stroke?
2. Which of the Kreuter and colleagues’ culturally appropriate strategies have been used in CBCTE programs for Black adults with cardiovascular disease, hypertension, diabetes, or stroke, and how have they been implemented in these programs?

### Methods

This systematic review was registered on the PROSPERO International Prospective Register of Systematic Reviews (CRD42021245772) and followed the detailed methods reported in the published protocol of this review [55]. Kreuter and colleagues’ five strategies for culturally appropriate interventions were used as a framework to evaluate and compare components of CBCTE programs included in this review. As described in Table 1, the five strategies outlined within this framework include (1) peripheral strategies (e.g., marketed to a target group), (2) evidential strategies (e.g., the inclusion of data/evidence to contextualize a health issue in a specific community), (3) linguistic strategies (e.g., language of intervention), (4) constituent-involving strategies (e.g., drawing on community members’ experience), and (5) sociocultural strategies (e.g., the inclusion of social or cultural values of target group within intervention) [57].

**Table 1:** Kreuter and colleagues’ five strategies for culturally appropriate interventions

Kreuter et al. culturally appropriate strategies [57]	Description of strategy
Peripheral	To convey the importance of the program/material to the target group through the appearance of cultural appropriateness
Evidential	To increase the perceived relevance of health topics using data (“evidence”) demonstrating the impact of specific health issues in the target community
Linguistic	To increase the accessibility of intervention materials by providing materials in the dominant or native language of the target group
Constituent-involving	To draw on community members’ experiences by involving them in an intervention
Sociocultural	Discussion of health issue/intervention material in the target group’s social context and cultural values, beliefs, and behaviors

#### Searches

The research team, including an information specialist (EMU), created a search strategy using medical subject headings (MeSH) and text words that related to the following concepts: (i) community-based; (ii) culturally tailored education; (iii) cardiovascular disease, hypertension, diabetes, or stroke; and (iv) Black adults. The following databases were searched on September 30, 2021: Medline, Embase (OvidSP), and Cumulative Index to Nursing and Allied Health Literature (EBSCOhost). The searches on these databases are included in the supplementary materials (Supplementary Material 1). In addition, a hand search of reference lists from 10 included studies was conducted to identify relevant articles that may have been missed.

#### Study Inclusion and Exclusion Criteria

Empirical full-length articles of any design and language were eligible if they were available in English, published on or after the year 2000, and reported the results of a CBCTE program for Black adults (18 years of age and older) who had cardiovascular disease, hypertension, diabetes, or stroke. To be considered a CBCTE program, the following criteria had to be met, as outlined in the protocol [55]:

• *Community-based:* must be delivered in community settings (e.g., participant’s home, a community clinic) and intend to share knowledge and experiences to develop a common understanding and require community members to have a role in the intervention (e.g., advisor, recipient) [58]
• *Culturally tailored*: must recognize a “group’s cultural values, beliefs and behaviours” by including at least one of the following five strategies: (1) peripheral; (2) evidential; (3) linguistic; (4) constituent-involving; or (5) sociocultural [57]
• *Education*: must deliver some form of education on managing hypertension, cardiovascular disease, diabetes, or stroke

#### Study Selection

The results of the database searches were imported into Covidence: a software used to deduplicate the search results and support study screening. After sufficient interrater reliability was achieved (kappa > 0.8), title and abstract screening and full-text review were conducted by in duplicate by two screeners (HS, JF, and/or a research assistant) to identify relevant articles based on the inclusion criteria. Any conflicts were resolved through discussions.

#### Study Quality Assessment

Two reviewers (MS, HS) independently critically appraised studies using one of the following Joanna Briggs Institute (JBI) Critical Appraisal Tools [59]: Checklist for Randomized Controlled Trials [60], Checklist for Cohort Studies [59], Checklist for Qualitative Research Studies [61], and Checklist for Quasi-Experimental Studies [62]. The JBI tools were selected because they offer a range of tools for various study designs and have been developed through an extensive peer review process [63].

#### Data Extraction Strategy

A research team member (SM) extracted data related to the study characteristics (e.g., year and country of publication, study design), setting, participants (e.g., health condition, age), intervention details (e.g., program name, duration), and outcomes (e.g., evaluation measures and intervention outcomes). A second reviewer (HS) reviewed all extracted data for accuracy. In addition, one reviewer (HS) extracted data related to the cultural tailoring strategies applied within included interventions [57].

#### Data Synthesis and Presentation

Extracted data relating to study characteristics, settings, participants, intervention details, and outcomes were descriptively reported in textual and table summaries. In addition, an inductive thematic analysis of each included study’s methods, results, and discussion completed by HS and JF was used to examine the application of cultural tailoring within programs [57, 64].

## Results

Seventy-four studies were included in this review (see Supplementary Material 2 for the Preferred Reporting Items for Systematic Reviews and Meta-Analyses flow diagram). Of the 74 studies included in this review, studies used qualitative (*n* = 5), quantitative (*n* = 59), and mixed (*n* = 10) methods. The three most common study designs were randomized controlled/clinical trials (51.4%), quasi-experimental/pre-post-test (16.2%), and pilot studies (9.5%). Most studies were published between the years 2016–2021 (*n* = 24/74; 32.4%) and 2011–2015 (*n* = 21/74; 28.4%), with fewer published in 2006–2010 (*n* = 15/74; 20.3%) and 2000–2005 (*n* = 14/74; 18.9%). See Table 2 for detailed study characteristics.

**Table 2:** Study characteristics

	Study design	Intervention structure	Intervention name/descriptor	Country
Cardiovascular disease				
Brewer et al. (2017) [49]	Cohort study	16 weeks (90-min education sessions biweekly)	FAITH! program	USA
Villablanca et al. (2016) [65]	Pre- and post-intervention study	8 group counseling sessions (90–120 min) every 2 weeks/4 months	Community-based educational cardiovascular program	USA
Diabetes				
Abbot et al. (2019) [51]	Cluster randomized controlled trial	3 sessions, 90 min–2 h each	Project POWER	USA
Anderson et al. (2005) [66]	Randomized controlled trial (pre-/post-test with repeated measures)	6 weeks (weekly 2-h sessions)	Problem-based empowerment program	USA
Anderson-Loftin et al. (2002) [67]	Longitudinal quasi-experimental study (1-group pre-/post-test)	4 classes, 6 discussion groups and follow-up phone calls and a home visit	Culturally competent dietary education	USA
Anderson-Loftin et al. (2005) [52]	Longitudinal experimental study (pre-/post-test control group)	4 weekly classes (1.5 h), 5 monthly group discussions, weekly phone call	Culturally competent dietary education	USA
Austin and Claiborne (2011) [68]	Case study, participant observation, focus group	Unclear	The Faith Wellness Collaboration, Project Power, Promoting Diabetes Education Program	USA
Bogner and de Vries (2010) [53]	Pilot randomized controlled trial	3 30-min in-person session, 2 15-min phone contacts over 4 weeks	Integrated care intervention	USA
Bray et al. (2005) [69]	Care management intervention (redesigned)	4 session group education/support over 6 months	Care management intervention	USA
Carter et al. (2011) [70]	Mixed methods (randomization + qualitative)	30-min biweekly 30-min video conferences	Diabetes telehealth self-management intervention	USA
Collins-McNeil et al. (2012) [71]	Mixed methods (pilot study)	Weekly 2-h sessions over 6 weeks + self-management practice for 6 weeks	Church-based culturally targeted (CBCT) diabetes self-management education (DSME) intervention	USA
Crowley et al. (2013) [72]	Randomized controlled trial (two-arm)	Monthly phone calls over a 12-month period	Cholesterol, Hypertension, And Glucose Education (CHANGE) study intervention	USA
Cummings et al. (2017) [73]	Post hoc analysis of the prospective randomized controlled trial	16 sessions	EPOWER study	USA
D'Eramo-Melkus et al. (2004) [74]	Pilot quasi-experimental study (one group, pre-/post-test)	6-weeks	Culturally competent intervention of education and care for Black women with type 2 diabetes mellitus	USA
Funnell et al. (2005) [75]	Randomized controlled trial	Six 2-h weekly group sessions and monthly follow-up (phone calls or support group meetings)	Empowerment-Based Diabetes Self-management Education Program	USA
Garvin et al. (2004) [76]	Mixed methods (evaluation study)	Regularly scheduled support group meetings, peer educator, educational classes, 6-week self-management classes, case coordination	Racial and Ethnic Approaches to Community Health (REACH) classes and support groups	USA
Gary et al. (2009) [77]	Randomized controlled trial	Minimal intervention: mailings and a phone call every 6 months; intensive intervention: 6 weeks	Project Sugar 2	USA
Gore et al. (2012) [78]	Evaluation study (pre-/post-test)	5 weeks	Recipe for health	USA
Han et al. (2019) [79]	Single-arm pre- and post-test (pilot study)	4-week literacy training and disease knowledge education, 2 home visits, monthly phone counseling for 24 weeks	PLAN 4 Success (Prevention through Lifestyle intervention And Numeracy)	USA
Hassaballa et al. (2021) [80]	Case study design (mixed methods)	12 months	Durham Diabetes Program	USA
Hendricks and Hendricks (2000) [81]	Pilot investigation	Monthly follow-up or 3-month intervals over 6 months	LHCA Diabetes Self-management Skills Training Center	USA
Keyserling et al. (2000) [82]	Randomized controlled trial	4 clinic visits, 12 monthly phone calls, 3 group sessions	New Leaf Choices for Healthy Living With Diabetes Program	USA
Keyserling et al. (2002) [83]	Randomized controlled trial	Clinic-based: 4 monthly nutritionist consults; community-based intervention: 4 monthly nutritionist consults and 3 group sessions and 12 monthly phone calls from peer counselor; minimal intervention: mailed pamphlets	Clinic and community (group A), clinic only (group B), or minimal intervention (group C); New Leaf Program	USA
Lachance et al. (2018) [84]	Evaluation study	Various programs; up to 10 weeks	National Kidney Foundation of Michigan (NKFM) Intervention Programs (various program names)	USA
Leeman et al. (2008) [85]	Pilot study	1-h-long 4 in-home sessions	Tailored diabetes self-care intervention	USA
Lutes et al. (2009) [86]	Randomized trial	16 phone-based lifestyle intervention sessions	Community health worker (CHW)–delivered lifestyle intervention; Empowering Rural African American Women and Communities to Improve Diabetes Outcomes (EMPOWER) trial	USA
Lynch et al. (2019) [87]	Randomized controlled trial	28 group sessions over 12 months (first 4 months-weekly; second 4 months-biweekly and third 4 months-monthly); two additional maintenance sessions month 15 and 18	Lifestyle Improvement through Food and Exercise (LIFE)	USA
Murrocket al. (2009) [88]	Pilot study (2-group pretest–posttest design; mixed methods)	12-week dance intervention-2 classes/week for 12 weeks	Dance and peer support	USA
Okoro (2020) [89]	Descriptive phenomenological study	Monthly peer support	Group-Based Peer Support Program	USA
Peek et al. (2012) [90]	Observational cohort pilot study	6 weekly sessions, 80–90 min long	Patient education intervention enhanced with positive-affect induction and self-affirmation	USA
Peña-Purcell et al. (2019) [91]	Pre-/post-test research design	7 weeks	Culturally tailored diabetes self-management education intervention	USA
Peña-Purcell et al. (2015) [92]	Prospective quasi-experimental, repeated measure	7 weeks	“Wisdom, Power, Control” diabetes self-management education	USA
Rovner et al. (2020) [93]	Randomized controlled trial	5 90-min sessions/3 months and 3 90-min sessions/9 months	OT behavioral intervention and the CHW-delivered diabetes self-management education intervention	USA
Rovner and Casten (2019) [94]	Pilot randomized controlled trial	6 months	Collaborative Care for Depression and Diabetic Retinopathy (CC-DDR)	USA
Ruggiero et al. (2014) [95]	Randomized controlled trial	Quarterly in-person 30-min sessions, monthly phone calls/1 year	Medical assistant self-care coaching (MAC) intervention	USA
Samuel Hodge et al. (2009) [96]	Randomized controlled trial	1 individual counseling visit, 12 group sessions, monthly phone calls, and 3 postcards, 4-month monthly phone calls	Church-Based Diabetes Self-Management Program	USA
Samuel Hodge et al. (2017) [97]	Randomized controlled trial	20-week group-based sessions offered > 2 times weekly, lasted 120 min, included a family time component (20-min)	Family PArtners in Lifestyle Support (PALS)	USA
Sharp et al. (2018) [98]	Randomized controlled trial	Monthly home visits and phone calls for 3–4 months, no. of visits and calls varied; community health worker and pharmacist communication	CHW supporting clinical pharmacists in diabetes management	USA
Skelly et al. (2005) [99]	Pilot randomized controlled trial	In-home, biweekly < 1 h; total 6 h	In-home, nurse-delivered, symptom-focused teaching/counseling intervention	USA
Spencer et al. (2011) [100]	Randomized controlled trial	2-h group sessions every 2 weeks, in-home visits, phone calls every 2 weeks	Community health worker intervention	USA
Steinhardt et al. (2015) [101]	Quasi-experimental design	8 weekly classes (2 h) + 2 biweekly support groups post-intervention	Resilience-based diabetes self-management education (RB-DSME)	USA
Tang et al. (2011) [102]	Pilot study	46 h of face-to-face group setting training sessions (2 times/week, 2 h/session) over 12 weeks	Peer-Led, Empowerment-based Approach to Self-management Efforts in Diabetes (PLEASED) intervention-diabetes self-management support (DSMS)	USA
Tang et al. (2005) [103]	Pilot study (longitudinal, prospective, predesign, and postdesign)	Weekly 90-min sessions over 6 months	Ongoing diabetes self-management support interventions	USA
Treadwell et al. (2010) [104]	Mixed methods, including qualitative (focus groups and interviews)	6 educational sessions (~ 12 h), fitness activities (~ 150 h), healthy eating, and lifestyle demonstrations (~ 30 h)	Save Our Sons (community-based, culturally responsive, gender-specific) intervention	USA
Two Feathers et al. (2005) [105]	Nonrandomized (pre-/post-test study)	5 2-h group meetings delivered every 4 weeks	Racial and Ethnic Approaches to Community Health (REACH) Detroit Partnership	USA
Two Feathers et al. (2007) [106]	Qualitative	5 2-h group meetings delivered every 4 weeks	Racial and Ethnic Approaches to Community Health (REACH)-Journey to Health	USA
Utz et al. (2008) [107]	Quasi-experimental design	Weekly group sessions (2 h each)/8 weeks; individual sessions (10–15 min) on 3 occasions/8 weeks	Group or individual diabetes self-management (DSME)	USA
Walker et al. (2010) [108]	Quasi-experimental design (pre-/post-test comparison group)	Three (2-h) interactive sessions	Self-management of diabetes program	USA
Whitney et al. (2017) [109]	Qualitative and pilot testing	8 classes	Patient Empowerment and Diabetes Education Curriculum	USA
Williams et al. (2014) [110]	Randomized controlled trial	8 2-h group sessions	“Taking Care of Sugar” DSME program	USA
Diabetes and hypertension				
Lynch et al. (2014) [111]	Pilot randomized controlled trial	6-month (18 3-h group sessions, weekly phone calls)	Lifestyle Improvement through Food and Exercise (LIFE) intervention	USA
Hypertension				
Bangurah et al. (2017) [112]	Pre- and post-intervention study	1 month (3 in-person meeting)	Nurse-led diet/exercise intervention	USA
Banks-Wallace (2007) [50]	Pre- and post-single group	12 months (3-h monthly meeting and in-home walking component)	Walk the Talk	USA
Beune et al. (2014) [113]	Cluster-randomized trial	3 30-min sessions of culturally appropriate counseling at weeks 2, 8, and 20; culturally appropriate written education; referral to lifestyle support	Culturally Adapted Hypertension Education (CAHE)	Netherlands
Bokhour et al. (2016) [114]	Randomized controlled trial (three-site)	1 time DVD viewing	DVD adding videos of African American Veterans telling stories about successful hypertension management	USA
Boutin-Foster et al. (2016) [115]	Randomized controlled trial (two-arm)	Phone calls every 2 months for 1 year	Trial using motivational interviewing, Positive Affect, and Self-Affirmation in African Americans with Hypertension (TRIUMPH)	USA
Brennan et al. (2010) [116]	Prospective randomized controlled intervention trial (stratified cluster randomization process used with the participant’s primary care physician office as the unit of randomization)	Monthly phone calls (15–20 min), mailed educational material	Telephonic nurse disease management (DM) program	USA
Greer et al. (2015) [78]	Randomized controlled trial	90-min weekly classes for 6 weeks	Culturally tailored intervention	USA
Gross et al. (2013) [117]	Evaluation study	8 weeks	Culturally sensitive education to improve anti-hypertension regimen adherence	USA
Liang et al. (2015) [118]	Randomized controlled trial	Phone calls 1, 3, and 5 months	Peer Coach and Office Staff Support Trial	USA
Marseille et al. (2021) [119]	Feasibility study (pre-/post-test)	45-min educational session	Evidence-based education program focused on improving knowledge, awareness, and management of hypertension	USA
Meinema et al. (2015) [120]	Cluster randomized controlled trial	Unclear	Culturally Appropriate Hypertension Education (CAHE) trial	Netherlands
Migneault et al. (2012) [121]	Randomized controlled trial (two-arm)	1 automated call/week for 32 weeks	Telephone-linked care	USA
Ogedegbe et al. (2012) [122]	Randomized controlled trial (two-arm)	Workbook, behavioral contract, bimonthly phone calls	Patient empowerment intervention	USA
Resnick et al. (2009) [123]	Feasibility study (single group, repeated measure)	12 weeks	People Reducing Risk and Improving Strength through Exercise, Diet and Drug Adherence (PRAISEDD)	USA
Schneider et al. (2005) [124]	Randomized controlled trial	TM program: twice/day for 20 min, brief interview, 1–1.5 h instructions, and 3 follow-up; sessions/3 days; PMR program: 15–20 min twice/day	Progressive Muscle Relaxation and Transcendental Mediation	USA
Schoenthaler et al. (2018) [125]	Cluster randomized controlled trial (two-arm)	11 90-min weekly group sessions, 3 monthly interviewing treatment sessions	Faith-based motivational interviewing therapeutic lifestyle change (MINT-TLC)	USA
Scisney-Matlock et al. (2006) [126]	Randomized controlled trial	30 days	Manage Associated Perceptions (MAP) of Dietary Behavior Study (Dietary Approaches to Stop Hypertension (DASH) diet)	USA
Thomas and Stoeckel (2016) [127]	Qualitative key informant study	1-h individual teaching sessions over 1 month, follow-up phone call after 1 week	Nurse protocol for medication self-administration	USA
Tully et al. (2015) [128]	Controlled clinical trial (pilot)	6 group sessions and weekly phone calls (6-month program)	Adjunct strategy to reduce blood pressure	USA
Victor et al. (2018) [129]	Cluster randomized controlled trial	Pharmacists met with participants at barbershop, prescribed and monitored medication regime and communicated with providers	Blood-pressure reduction in Black barbershops	USA
Victor et al. (2019) [130]	Cluster randomized controlled trial	Unclear	Blood pressure reduction in Black barbershops	USA
Webb et al. (2006) [131]	Pilot randomized controlled trial	6 weekly (1 h) classes + 4-week self-practice	Cognitive-mediation and relaxation interventions	USA
Stroke				
Sajatovic et al. (2018) [54]	Prospective randomized controlled trial and qualitative interviews	60-min 1:1 initial session, 4 60-min group sessions, 7 10–20-min phone sessions/6 months	TargetEd MAnageMent Intervention (TEAM)	USA

### Risk of Bias

Seventeen studies were not critically appraised due to an unclear study design, or their study design did not align with the JBI checklists (e.g., evaluation, feasibility, pilot studies). The average critical appraisal score of randomized control trials was 7.2/13 (range 1–12; SD: 2.5), with variations in study quality related to factors such as not clearly describing the randomization procedure, participants in the control and treatment groups not being similar at baseline or having different treatment other than intervention, unclear follow-up procedures, and not including intent-to-treat. The average critical appraisal score of quasi-experiments was 5.3/9 (range 3–9; SD: 2.2), with variations in study quality related to factors such as not having a control group and reliable measurement of outcomes. The average critical appraisal score of cohort studies was 5/11 (no range; SD: 0), with variations in study quality related to factors such as participants not being free of the outcome at the start of the study and unclear descriptions of follow-up and strategies used to manage incomplete follow-up. The average critical appraisal score of qualitative studies was 7/10 (range 6–8; SD: 0.7), with qualitative studies unclearly reporting the researchers’ cultural or theoretical stance and failing to address how the researcher influenced the research.

### Research question 1: What are the program characteristics and outcomes of CBCTE programs designed to improve health outcomes in Black adults with cardiovascular disease, hypertension, diabetes, or stroke?

### Program Characteristics

*Location (country):* Nearly all programs were conducted in the USA (72/74; 97.3%), while the remaining 2 (2/74; 2.7%) were conducted in the Netherlands.

*Location (Site):* Thirteen programs (13/74; 17.6%) were delivered in community settings, but the type of community site was unclear. Of the remaining 61 programs, 39 (39/74, 52.7%) were delivered exclusively within a single site (i.e., a church (8/39; 20.5%), a participant home via home visit, online or telephone (14/39; 35.9%), community center (5/39, 12.8%), health center/clinic (10/39; 25.6%), and barbershop (2/39; 5.1%)). The other 22 (22/74; 29.7%) programs were delivered in multiple sites (e.g., health centers/clinics and participant’s homes (11/22; 50.0%), churches and community centers/facilities (3/22; 13.6%), church and participant’s home (1/22; 4.5%), different community locations (4/22; 18.1%), or community locations and participant’s home (3/22; 13.6%)).

*Study duration:* The duration of studies ranged from ≤ 6 months (42/74; 56.8%), > 6 months to ≤ 1 year (8/74; 10.8%), and > 1 year (21/74; 28.4%), while 3/74 (4.05%) had an unclear duration.

### Participant Characteristics

*Health conditions targeted:* The majority of programs targeted diabetes (48/74; 64.9%) and hypertension (22/74; 29.7%), while 1/74 (1.3%) targeted both. Other targeted conditions included cardiovascular disease (2/74; 2.7%) and stroke (1/74; 1.3%). See Supplementary Material 3 for sample characteristics.

*Target ethnic groups of programs:* Most programs targeted exclusively Black/African American program participants (61/74; 82.4%), with some studies targeting African Americans and other ethnic groups (e.g., Hispanics/Latinos, Native Americans, Asians) (10/74; 13.5%). A few studies targeted “Haitian Immigrants” (1/74; 1.3%) and “African Surinamese and Ghanaian” participants (2/74; 2.7%).

*Sample sizes:* The total sample size of the programs ranged from 9 to 638.

*Sex and age-targeted:* While most programs targeted both men and women (53/74; 71.6%), some targeted only females (15/74; 20.3%) or males (6/74; 8.1%). A majority of programs had an inclusion criterion for adults of 18–30 years (33/74; 44.6%), while others had criteria for mid-adult (35 years or older) to older adult ages (18/74; 24.3%), or the targeted minimum age was unclear (23/74; 31.1%).

### Program Outcomes

Table 3 reports examples of outcome measures used within included studies by category. Various outcome measurements were used to evaluate interventions, including participant-level (e.g., health-related outcomes, health literacy, medication adherence, psychosocial measurements) and program-specific outcome measures (e.g., program adherence, satisfaction). Most studies (*n* = 50/74; 67.6%) examined a type of physiological outcome measure (e.g., hemoglobin A1C, weight/body mass index); however, health care satisfaction, involvement, disparity, and use were rarely measured (*n* = 8/74; 10.8%). In addition, some participant-level outcome measures were culturally tailored to community members. For instance, researchers used outcome measures adapted for use with African Americans (e.g., Community Healthy Activities Model Program for Seniors physical activity questionnaire modified for African Americans and an adaption of Food Habits Questionnaire for participants) [52, 111]. Others reported that the outcome measure was reliable and validated for use by this community [94, 119]. See Supplementary Material 4 details the outcome measures used in each study.

**Table 3:** Examples of outcome measures used within included studies

Category measured	Examples of outcome measures administered
Physiological, *n* = 50/74, 67.6%	Blood pressure (rate); height/weight/body mass index; hemoglobin A1C; cholesterol or lipid profile (e.g., low- and high-density lipoprotein cholesterol)
Self-management, problem-solving, *n* = 29/74, 39.2%	Diabetes Self-Care Inventory–Revised; The Summary of Diabetes Self-Care Activities; Diabetes Self-Care Practices Measurement Questionnaire; 7 Self-Care; Behaviors Tracking; customized form; blood pressure monitoring frequency, Hill-Bone Compliance to High Blood Pressure Therapy Scale; Diabetes Management Practices Scale; Self-Appraisal Diabetes Self-Management Scale; Self-Care Inventory-Revised; Diabetes Quality Improvement Project; cardiovascular risk; physical activity and dietary practices through questions from the Centers for Disease Control’s Behavioral Risk Factor Surveillance System. Glucometer use
Medication-related (e.g., adherence, change, knowledge, and beliefs), *n* = 20/74, 27.0%	Electronic pill monitor; Morisky Medication Adherence Scale; Medication Adherence Self-Efficacy Scale; Cardiac Medication Adherence Outcome Expectation Scale; Number of antihypertension medication classes; Beliefs about Medicines Questionnaire; decisional conflict regarding antihyperglycemic medication, helpfulness and clarity of the medication information
Self-efficacy, resilience, empowerment and coping, fatalism, *n* = 15/74, 20.3%	Diabetes Care Self-Efficacy Scale; Diabetes Empowerment Scale-Short Form; Diabetes Self-Efficacy Outcomes Expectancies questionnaire; decision self-efficacy scale; Connor-Davidson Resilience Scale; Positive adaptation; Coping Orientations to Problems Experienced Scale; Diabetes Fatalism Scale; Personal Resource Questionnaire
Diabetes knowledge and literacy, *n* = 16/74, 21.6%	Diabetes Knowledge test; Diabetes Knowledge Questionnaire; Customized Diabetes Knowledge Test; Spoken Knowledge in Low Literacy in Diabetes Scale; New Leaf Diabetes Knowledge Instrument; Perceived Competence for Diabetes scale
Psychological, *n* = 15/74, 20.3%	Center for Epidemiologic Studies Depression Scale, Epidemiological Studies Depression Scale; Patient Health Questionnaire; Stress/distress or Anxiety Symptoms; Kessler-6 Questionnaire
Diet, *n* = 13/74, 17.6%	Minnesota Nutrition Data System; Monitoring sodium intake; Alternative Healthy Eating Index; Food Habits Questionnaire; Diet Habit Survey; Healthy Eating Scale; Block Food Frequency Questionnaire, Nutrition Data System for Research 24-Hour Dietary Recall; Nutritional Knowledge Questionnaire; Food Frequency Questionnaire; Self-efficacy for Health-Related Diet; A Single-item Diet Outcome Expectations Measure; adapted Lifestyle Cognitive Representations Scales; Sodium Intake Behaviors
Health knowledge and literacy, *n* = 12/74, 16.2%	Newest Vital Sign; Hypertension Knowledge Test; High blood pressure Prevention IQ, Revised Diabetes Knowledge Test
Quality of life, *n* = 13/74, 17.6%	Short Form (SF)-12/SF36; Euro Quality of Life; Diabetes Care Profile; Social and Personal Factors subscale from Diabetes Care Profile; Problem Areas in Diabetes; Stroke Impact Scale
Physical activity, *n* = 10/74, 13.5%	Physical activity scale; International Physical Activity Questionnaire; 7-day physical activity, exercise behavior; Physical Activity Questionnaire; pedometer; Physical activity self-report; Yale Physical Activity Survey; Community Health Activities Model Program for Seniors; Physical activity questionnaire modified for use by African Americans; Level of Exercise Stages of Change; Paffenbarger Physical Activity Questionnaire; Cross-Cultural Activity Participation Study; self-efficacy for Exercise Scale; Outcome Expectations for Exercise scale; Problem Areas in Diabetes Survey Exercise Benefits/Barriers Scale; pedometers
Self-perceived health, *n* = 8/74, 10.8%	Brief Illness Perceptions Questionnaire; Perceptions of hypertension; custom questionnaires
Social support or personal resources, *n* = 8/74, 10.8%	Duke Social Support Scale; Medical Outcomes Study Social Support Survey; modified Medical Outcomes Study-Social Support Survey; Support Received subscale from Diabetes Care Profile; Chronic Illness Resources Survey; Personal Resources Questionnaire
Healthcare use/access, *n* = 6/74, 8.1%	Healthcare utilization questionnaire; Follow-up rates; Number of primary care visits; National Health Information; Cost of care questionnaire; Access to care questionnaire
Functional, *n* = 5/74, 6.8%	Balance, Gait, Grip strength
Attitudes/outlook, *n* = 3/74, 4.0%	Diabetes Attitude Scale; Outlook on life and health status
Healthcare satisfaction, involvement, disparity, *n* = 2/74, 2.7%	Consumer Quality Index-Diabetes; Patient and Provider Shared decision-making Behaviors; Health Disparity Issues Ranking
Addiction, *n* = 2/74, 2.7%	Addiction Severity Index; Fagerstrom Test for Nicotine Dependence
Mood, *n* = 2/74, 2.7%	State-Trait Anger Expression Inventory; Positive and Negative Affect Schedule; World Health Organization -5-wellbeing index
Program-specific outcomes	Program attendance/Retention, Acceptability, Satisfaction and Feasibility surveys/interviews; Focus groups, Satisfaction with Diabetes Education Program, ‘Taking Care of Sugar (satisfaction with diabetes education program)

Positive changes were reported by studies on outcome measures, including physiological (e.g., [65, 66, 74, 90, 97, 101, 113, 130]), medication-related (e.g., medication adherence, medication change, medication knowledge and beliefs; e.g., [72, 73, 87, 98, 113, 129]), physical activity (e.g., [50, 54, 83, 112]), quality of life (e.g., [99, 103]), psychological (e.g., [53, 97]), diabetes knowledge and literacy (e.g., [99, 101, 110]), health knowledge and literacy (e.g., [49, 51]), self-efficacy (e.g., [91]), self-management (e.g., [100, 110]), resilience/empowerment (e.g., [66, 73]), healthcare use, (e.g., [130]), diet (e.g., [111, 121]), and self-perceived health (e.g., [120]). Some studies indicated that study limitations contributed to a lack of intervention impacts, such as a short intervention duration providing insufficient participant contact to improve outcomes [49, 51, 78] and a small sample size [49, 54, 69, 78, 79, 88, 96, 97, 101, 108, 110, 112, 113, 131, 132].

### Research question 2: Which of the Kreuter and colleagues’ culturally appropriate strategies have been used in CBCTE programs for Black adults with cardiovascular disease, hypertension, diabetes, or stroke, and how have they been implemented in these programs?

Table 4 categorizes cultural appropriateness strategies used within studies by the Kreuter et al. framework [57].

**Table 4:** Cultural appropriateness strategies used within studies categorized within the Kreuter et al. framework

Kreuter et al. strategies of culturally appropriateness	*Example of how specific strategies were employed within CBCTE programs for Black communities
Peripheral strategies	-marketed as a culturally specific program for Black/African Americans (all included studies) -cookbooks designed for African Americans [68] -showed videos, cases, stories, or vignettes of African American individuals (e.g., lay individuals and key celebrities such as Yolanda King) sharing information about the target condition, management strategies, and health challenges to share relatable examples [65, 78, 90, 114] -incorporated voice messages [114], images, illustrations, and videos of African American individuals engaging in target behaviors (e.g., hypertension management; peer-modeling) [74, 80, 106, 114] -shared letters containing spiritual messages with participants [49] -incorporated symbols or themes into intervention content [106]
Evidential strategies	-use of the African American Health and Dairy Foods Nutritional Fact Sheet, 7 Steps to a Healthy Heart for African Americans [116], National Diabetes Clearinghouse article Diabetes in Black Americans, Black Quilt Cookbook [74], American Heart Association’s (AHA) “High Blood Pressure in African Americans” [119] -discussion of race-specific health strategies (e.g., foot care for the skin of color) [74]
Linguistic strategies	-use of local providers for intervention delivery to minimize linguistic barriers and align with the dialect and accent of the target community (e.g., rural southern accent, vernacular, and idioms) to foster trust between intervention provider and recipient [67]
Constituent-involving strategies	-showed a video of African American individuals (e.g., lay individuals and key celebrities such as Yolanda King) sharing information about the condition and management strategies [78, 114] -presentations conducted by racially concordant research team members and health providers [68, 71] -partner with community members or advisors (e.g., lay/peer/community health workers/community diabetes advisor/liaisons, church pastors, local Black leaders, community members) to develop (e.g., get feedback on), deliver, and evaluate the intervention; race-concordant providers involved in intervention delivery [49, 51, 52, 54, 65, 67, 68, 71, 73, 74, 76–79, 82–88, 91, 93–95, 98, 100–102, 104–106, 108, 109, 111, 114, 118, 119, 124, 125, 128–130, 132] -use of race-concordant assistants if there is a lack of race-concordant providers available for intervention delivery [67, 78] -partner with organizations that serve African Americans (e.g., churches, and health organizations) using community-based participatory action research or community-engaged approaches to ensure cultural congruence of planned intervention [68, 79] -qualitative investigations and literature reviews were conducted pre-intervention to inform intervention design and delivery [49, 68, 85]
Sociocultural strategies	-cookbooks designed for African Americans (e.g., Soul Sensation Cookbook) [68, 74] -shared letters containing spiritual messages with participants [49] -dance classes choreographed to gospel music [88] -use of cultural recipes (e.g., Soul Food) [74] -discussion of cultural reinforcements and barriers to health management (e.g., dietary modification, diabetes-related self-care) [74, 90] -educational materials posted in the church to reinforce education [132] -nutritional instructions/education aligned to the target group’s shopping and dietary patterns [90] -intervention advertised or delivered in faith-based organizations (e.g., churches) and community organizations serving the target population [65, 76, 78, 96] -involvement of family members in the intervention [67, 90, 97, 107] -incorporate oral transition of storytelling and religious tradition of testifying [90] -inclusion of prayer and spiritual messages with participants [49] -delivering in barber shops [129, 130] -social determinants (e.g., income, literacy level) [52, 66, 70, 87, 96, 123]

^*^Some examples fit within more than one strategy

All studies within this review employed peripheral strategies because our inclusion criteria required studies to report a CBCTE program for Black communities (i.e., marketed to Black adults). Evidential strategies included incorporating resources developed for the target community within the intervention [131] (e.g., 7 Steps to a Healthy Heart for African Americans [116]).

Constituent-involving strategies involved members of Black communities and community organization representatives serving these communities. Pastors, spiritual counselors, and other community leaders were viewed as gatekeepers to accessing the target communities, and they helped ensure that the programs aligned with the target groups’ cultural practices (this overlaps with sociocultural strategies) while also being feasible and acceptable to the target communities [71]. Various terms were used to describe constituents: peer leaders [102], community health workers [77, 84, 86, 93, 94, 98, 100, 104, 128], family health advocates [100], trained community residents [105, 106], barbers [129, 130], community site leaders [65], patient navigators [108], local African American residents who were dietician assistants [67], church cook volunteers [68], African American veteran [114], liaisons [49], local pastors/clergy [71], peer advisor [73], lay health assistants [74], lay or peer leader [76, 102, 132], peer educator [76], research assistants (local African American women) [78], dance instructor [88], church diabetes advisor (peer counselor) [96], faith community leaders [79], community diabetes advisor [82, 83], peer coaches [118], peer supporter [111], class leaders [91], peer dyads [54], African American instructor [124], and lay health advisor [125]. Others involved “community advisory boards” (e.g., organization representatives that serve the target community, African American women) [85, 128, 132]. Some authors specified that constituents received training [54, 65, 76, 80, 91, 94, 98, 100, 102, 104–106, 111, 118, 125, 130, 132] (e.g., “cultural competent information, training and skill-building activities” [132]; “CHW received extensive training (50 hours) [86]), with some using train-the-trainer approaches [132]. Constituents were trained by assigning readings and use of training cases.

Constituents’ roles included intervention assessment, design/development/planning (e.g., develop/inform recruitment strategy [125, 128], approving educational materials [132]), implementation/delivery (e.g., providing education [106], accompanying participants to medical visits [100], provide guidance to address study barriers [128]), evaluation (e.g., review study progress [128]), and “data-sense making and program adjustments to address gaps” [80]. In addition to supporting intervention development and delivery, constituents supported intervention refinement [84, 100, 125], fostered participant buy-in to the intervention, and ensured intervention alignment with the cultural preferences of the community [112]. Race-concordant health providers delivered some interventions; however, when race-concordant providers were not available, the providers received training on the target population’s culture or had prior experience working with the target community [52, 53, 116]. Some also recruited race-concordant community members (e.g., research assistants/patient partners) to support race-discordant health providers with intervention delivery [67, 73, 87, 90, 91].

Translation of intervention materials to a different language was not apparent in any of the interventions. However, linguistic strategies included recruiting local community members to ensure that the intervention was delivered in the dialect and accent of the target community (e.g., rural southern accent, vernacular, and idioms), which is believed to foster trust between the intervention provider and the recipient [67]. Other linguistic strategies included having materials delivered by a Black community member (e.g., voice recording by an African American adult) [52, 121]. Some studies also integrated the community’s religious practices into the program communication; this strategy also overlapped with sociocultural strategies [71, 78]. For example, one study indicated that they opened and closed the sessions with prayer, and activities included reading scriptures [71]. In addition, some studies tailored to target participants’ social determinants of health, such as literacy levels (e.g., by simplifying intervention materials) [52, 90, 109, 110].

Sociocultural strategies involved intervention logistics, such as the delivery format, location, and program timing. For instance, virtual delivery facilitated participation and retention by removing barriers, such as transportation (e.g., costs) and competing roles priorities (e.g., caretaking responsibilities). Locations such as churches and barber shops were perceived as ideal locations to reach these communities as these were commonly attended locations and were seen as cultural hubs [65, 76, 78, 129]. In terms of program timing, flexible schedules during weekends and evenings accommodated work schedules and reduced participation barriers. Finally, sex-based differences should be considered in chronic disease as the authors indicated that their samples included a greater proportion of females [70] and recommended oversampling for males given poorer chronic disease management (e.g., blood pressure control) [115]. Sociocultural strategies also consisted of the inclusion of gospel music [88], spiritual messages with participants [49], incorporating oral transition of storytelling and religious tradition of testifying [90], and prayer and spiritual messages [49].

Other social determinants of health were considered in CBCTE programs. For instance, researchers restricted their inclusion to a specific education level (e.g., ≥ eighth grade) while noting that this education level might not be attained by all target community members, such as those in lower socioeconomic strata or older individuals, but it was required as the intervention was delivered online and comprised of reading materials [70]. Some studies specifically targeted individuals with low income [87, 95, 98, 123]. Finally, researchers individually tailored some interventions to intervention components relevant to each participant’s specific needs, preferences, diet, goals, medication adherence, barriers, and life experiences to effectively address multiple intersecting factors influencing participants’ needs [53, 72, 74, 75, 77, 79, 85, 95, 99, 110, 111, 115, 117, 123, 131].

## Discussion

This systematic review provides insights into the characteristics of CBCTE programs for Black adults with diabetes, hypertension, cardiovascular disease, and stroke. More studies have been published each year since 2000 (e.g., 19% of articles included in this review were published between 2000 and 2005 compared to 32% in 2016–2021). This finding may be a product of more attention and interest in health disparities in the USA [133]. The Kreuter and colleagues framework helped identify and categorize the various strategies used to tailor CBCTE programs to Black communities with cardiovascular disease, hypertension, diabetes, or stroke. These strategies can be adopted by existing and future programs seeking to culturally tailor. The strategies align with many of the approaches identified in a review by Wadi and colleagues in which the Facilitator-Location-Language-Messaging (FiLLM) framework was used to identify the cultural tailoring of methods in diabetes programs for Black communities. Their review suggested that the following aspects of programs were tailored: the materials (e.g., messages, written materials), location (e.g., churches), and constituents (e.g., community members) [31]. Our review extends findings from Wadi and colleagues as we examined programs beyond diabetes, including programs that target cardiovascular disease, hypertension, and stroke management. Interestingly, prior literature indicates that tailoring multiple components resulted in more effective interventions [31]. We highlight the variability of strategies used within the CBCTE program, especially with sociocultural strategies, which can overlap into multiple categories.

Based on reported results from the included studies, we noted that CBCTE programs have beneficial outcomes, such as improving diabetes knowledge and literacy [99, 101, 110], health knowledge and literacy [49, 51], self-efficacy [91], and self-management [100, 110]. Moreover, researchers reported small sample sizes as a limitation that may have caused some insignificant program impacts [49, 54, 69, 78, 79, 101, 108, 110, 112, 131, 132]. This finding is consistent with previous literature on culturally tailored health interventions [43, 47, 48].

Our analysis noted two critical knowledge gaps that should be addressed in future research. First, we noted that contextual knowledge gaps exist since most programs were conducted in the USA. This finding resonates with a systematic review of diabetes interventions tailored to communities of Black African ancestry [31]. Since Black communities experience health disparities in other countries (e.g., UK [134] and other European countries [135, 136], and Canada [137]), more research from locations beyond the USA is needed to account for possible contextual differences (e.g., differences in public and private health systems/resources). Second, we found a limited number of CBCTE programs for managing stroke and cardiovascular conditions, as most CBCTE programs included in our review targeted diabetes or hypertension. This gap should be addressed in future interventions as Black communities are at a greater risk for stroke [138–140] and cardiovascular disease [141–143], experience worse outcomes [144, 145], and have lower use of health services than other ethnic groups [146–148]. Three additional insights from our review are discussed in detail below.

*Insight 1: Unclear Roles and Degrees of Involvement of Communities*

There were varying community member roles and unclear degrees of community member involvement within interventions. The included studies failed to report community members’ roles, decision-making processes, and community member training consistently and comprehensively. For instance, some researchers adapted existing programs, whereas others designed programs with members of the communities.

Involving members of the communities and community-based organizations in the design and delivery was a common strategy in CBCTE programs. This finding is unsurprising as this strategy has been widely used to promote health research participation for Black communities by reducing participation barriers such as mistrust, continuing discriminatory events, and staff competence [149–153]. Including community members in health research can also foster participants’ trust in the research/researchers, help create culturally appropriate designs and community ownership, improve participant outcomes, and increase participants’ self-efficacy and empowerment [150, 154]. Moreover, community-based organizations that serve the target populations can advocate on the community’s behalf to ensure programs align with communities’ needs [155].

However, community members’ involvement in research tends to be poorly defined [150]. This finding is problematic because traditional top-down research approaches may not be effective with ethnically marginalized communities as they may not be conducive to shared decision-making and can fail to honor the expertise of community members [150]. Moreover, unclear descriptions of community members’ involvement can limit the replicability and evaluation (e.g., tokenism) of CBCTE programs. For instance, it is unclear whether/how community members’ perspectives were meaningfully included, whether/how they were adequately trained, and decision-making processes when perspectives contradicted the researchers [155]. Future research should critically reflect on and transparently report how community members can be meaningfully involved throughout the design process [155]. Moreover, evaluating the impact of community members’ involvement on community and individual-level outcomes is necessary to ensure continued dedication in future research [155, 156]. This finding begs the question: What are the hallmarks of meaning engagement?

CBCTE programs have integrated community members’ perspectives using various approaches, including codesign. Codesign, often used interchangeably with coproduction and community or patient engagement, has gained popularity [155]. However, researchers encourage future interventions to critically reflect on whether community members are meaningfully involved in research [155]. Moreover, when working with historically marginalized communities, researchers must address power differences to facilitate a safe and inclusive collaborative space and be open to perspectives that diverge from the status quo [149, 155]. *Design justice* is an approach that can be used to create CBCTE programs to attend to these factors because it challenges structural inequalities, considers the complex relationship between design, power, and social justice, and enables marginalized communities to lead the design [157].

*Insight 2: Program Location Matters*

The location appeared to be a critical consideration in CBCTE programs. CBCTE programs were often conducted in single sites (e.g., a participant’s home or the church) and, less commonly, health clinics. Health clinics may not be as common location for CBCTE programs given that Black communities tend to have lower use of primary care than White adults for many complex reasons, including medical mistrust and experiences of healthcare discrimination [158, 159]. Members of Black communities are more likely than other ethnic groups to attend church [160–162]. Integrating religiosity and social support/structure from the church is a well-known way to build trust in Black communities [149]. Due to churches’ valuable role and influence on Black communities, there has been a growing demand for engaging churches in health promotion and education interventions to reduce health disparities [149, 163–166]. However, researchers delivering single-site programs must consider which groups are underrepresented, as there is heterogeneity and varying needs among Black communities and intersectionality considerations must be considered. For instance, Black men may be less religious as they are found to have lower rates of church attendance than Black women [167, 168]. Sex differences in church attendance may explain why church-based CBCTE programs had a higher proportion of female participants, despite Black males having higher chronic disease presence and poorer prognosis than other ethnic groups [138–143].

One study was conducted in a barbershop to reach Black men [130]. Barbershops have been leveraged for health education and promotion interventions because it is a staple “in the African American community” [149, 169]. Barbershops are attractive sites for health interventions because they are culturally appropriate, and community members may stay for hours per visit to socialize and engage in productive activities, such as selling and advertising products [169]. The owners are often included in the interventions as they tend to be respected and influential community members [149]. Men attend barbershops, whereas their equivalent is hair salons attended by women [149]. While no CTCE programs included in this review were conducted at hair salons, other health interventions have been conducted at a hair salon (e.g., breast cancer [170–172]). Although barbershops and hair salons show promise for health interventions (e.g., cancer screening and hypertension management), more research is needed to ensure such interventions are acceptable and sustainable [169].

A third (30%) of CBCTE programs included in this review were conducted in multiple locations (e.g., church and community centers). Delivering interventions in multiple sites may increase the representativeness of various groups. Therefore, an implication is that researchers carefully consider which location site they will deliver the program and report the characteristics of individuals who are not included in their interventions. It is also worthwhile to explore the characteristics of community members recruited from each site location and test/evaluate whether program outcomes differ based on the delivery site.

Sustainability data can help determine the continued benefits and maintenance of community partnerships [173] and at the individual level (e.g., long-term impact of the program on patient outcomes) [174]. However, few studies included in this review evaluated intervention sustainability. For instance, Victor and colleagues found that blood pressure reduction was sustained in education provided by Black barbers [130]. However, more research is needed to evaluate CBCTE interventions' sustainability and cost-effectiveness to support informed decisions about program sustainability [48].

*Insight 3: Individualizing Culturally Tailored Interventions *t*oto Address Social Determinants of Health and Culture as Dynamic and Experienced Differently by People*

I﻿n addition to culturally tailoring CBCTE programs to the needs of Black communities, many researchers also individualized the programs to participants’ needs (e.g., literacy level, economic status). Individual-level tailoring approaches consider group heterogeneity and intersectionality, the latter referring to the multiple identities and experiences that a person has, such as race/ethnicity, gender, age, and migration status, that can impact disparities [175, 176]. Heterogeneity and intersectionality of a group/community may also require individualization beyond or integrated alongside group-based cultural tailoring approaches [176]. The purpose of cultural tailoring (population level) is to adapt the “study design, materials and other components of the intervention to reflect cultural needs and preferences at the population level” [35]. In contrast to population-level cultural tailoring, individualization involves creating program content “based on individuals’ existing behaviors, stages of behavior change, preferences, barriers, and other recognizable features interventions” [177, 178]. Individual-level tailoring seemingly addressed participants’ social determinants of health and individual preferences, such as their health literacy, individual diet, and program schedule. Individual-level tailoring and cultural tailoring to specific groups may be needed to address the needs of our increasingly diverse populations and the intricacies of Black cultures, which is not homogenous [179]. Additionally, given the variability noted in this review, future research is needed to examine the process and impact of variable representations of race and culture within research.

## Strengths and Limitations

This review has some limitations. First, as with all reviews, there is a risk of missing relevant studies. Relevant studies could have been inadvertently missed for reasons such as our search strategy or data screening errors. To reduce the risk of missing relevant studies, we created the search strategy in consultation with an experienced librarian, searched multiple databases, screened titles, abstracts, and full texts in duplicate, and performed a hand search based on the reference lists of included studies. Second, there is a risk of data errors during extraction. Two reviewers were involved in data extraction to reduce the risk of data error (one extracting and the second checking accuracy). Third, we limited the inclusion to empirical studies, excluding development studies and protocols. Some details about CBCTE programs’ design and delivery may have only been reported in the protocol paper and not in the empirical papers. Fourth, we did not perform a meta-analysis, given the heterogeneous study designs and outcome measures used within the included studies [47]. However, we could provide some insights into program outcomes based on results from the included articles. Fifth, this review is limited to CBCTE programs within community-based settings, and programs in other settings require further investigation. Finally, these findings must be interpreted with caution as they are based on studies which vary in methodological quality, based on our critical appraisal using the JBI critical appraisal checklists.

## Conclusion

This systematic review provides insights into the characteristics and cultural tailoring strategies used within culturally tailored community-based education interventions for Black adults with diabetes, hypertension, cardiovascular disease, and stroke. Several gaps and insights were revealed in this review. These findings can inform the modification of existing or development of new programs.

### Supplementary Information

Below is the link to the electronic supplementary material.

Supplementary file1 (DOCX 24 KB)

Supplementary file2 (DOCX 50 KB)

Supplementary file3 (DOCX 35.1 KB)

Supplementary file4 (DOCX 29 KB)

## Data Availability

The data analyzed in this study were from published articles, and are available from listed databases.

## Abbreviations

CBCTE: Community-based culturally tailored education
MeSH: Medical subject headings
PRISMA: Preferred Reporting Items for Systematic Reviews and Meta-Analyses
