# Supplementary material for: Community-Based Culturally Tailored Education Programs for Black Communities with Cardiovascular Disease, Diabetes, Hypertension, and Stroke: Systematic Review Findings
Source: J Racial Ethn Health Disparities. 2022 Dec 12;10(6):2986–3006. doi: 10.1007/s40615-022-01474-5 (PMC10645635; doi:10.1007/s40615-022-01474-5)
Supplement: Supplementary file 1 — Supplementary file1 (DOCX 24 KB) [file 40615_2022_1474_MOESM1_ESM.docx]

Supplementary material 1: Full Searches

**OVID MEDLINE**

| **#** | **Searches** | **Results** |
| --- | --- | --- |
| 1 | african continental ancestry group/ or african americans/ | 91690 |
| 2 | (african adj2 american*).ti,ab,kf. | 51564 |
| 3 | or/1-2 | 112373 |
| 4 | Culturally Competent Care/ | 1887 |
| 5 | cultural diversity/ or Cultural Characteristics/ | 28371 |
| 6 | cultural competency/ | 6048 |
| 7 | (cultural* adj2 adapted).ti,ab,kf. | 1661 |
| 8 | (cultural* adj2 competen*).ti,ab,kf. | 5244 |
| 9 | (cultural* adj2 tailor*).ti,ab,kf. | 1216 |
| 10 | or/4-9 | 39177 |
| 11 | self care/ or blood glucose self-monitoring/ or self administration/ or self medication/ | 57615 |
| 12 | exp Cardiovascular Diseases/ | 2528700 |
| 13 | glucose metabolism disorders/ or exp diabetes mellitus/ or exp glycosuria/ or exp hyperglycemia/ or exp hyperinsulinism/ or exp hypoglycemia/ | 554001 |
| 14 | aftercare/ or exp rehabilitation/ | 3237982 |
| 15 | or/11-14 | 292 |
| 16 | 3 and 10 and 15 | 431544 |
| 17 | attitude to health/ or health knowledge, attitudes, practice/ or "treatment adherence and compliance"/ or "patient acceptance of health care"/ or patient compliance/ or medication adherence/ or no-show patients/ or patient dropouts/ or patient participation/ or patient satisfaction/ or patient preference/ or treatment refusal/ or vaccination refusal/ | 173976 |
| 18 | consumer health information/ or health literacy/ or health promotion/ or healthy people programs/ or weight reduction programs/ or patient education as topic/ | 377 |
| 19 | 3 and 15 and 17 and 18 [**Base set 7 Attitudes and Ethnic Groups and Diseases and patient education**] | 641 |
| 20 | 16 or 19 | 627 |

**Ovid MEDLINE(R) Epub Ahead of Print**

| **#** | **Searches** | **Results** |
| --- | --- | --- |
| 1 | (african adj2 american*).ti,ab,kf. | 1318 |
| 2 | (cultural* adj2 (divers* or character*)).ti,ab,kf. | 179 |
| 3 | (cultural* adj2 adapted).ti,ab,kf. | 114 |
| 4 | (cultural* adj2 competen*).ti,ab,kf. | 244 |
| 5 | (cultural* adj2 tailor*).ti,ab,kf. | 81 |
| 6 | or/2-5 | 582 |
| 7 | (self adj2 (care or monitoring or administration or medication)).ti,ab,kf. | 1166 |
| 8 | ((cerebro* or cerebral* or myocardial or heart or cardiac or Cardiovascular or vascular) adj2 (Disease* or disorder*)).ti,ab,kf. | 7942 |
| 9 | (hypertensi* or infarct or infarction or stroke or strokes).ti,ab,kf. | 13894 |
| 10 | ((glucose adj2 metabolism adj2 (disorder* or disease*)) or (diabetes or diabetic or glycosuria or hyperglycemi* or hyperinsulini* or hypoglycemi*)).ti,ab,kf. | 11437 |
| 11 | (aftercare or rehab*).ti,ab,kf. | 11515 |
| 12 | or/7-11 | 5315 |
| 13 | 1 and 6 and 12 | 33387 |
| 14 | ((attitude* adj2 (health or knowledge or practice or compliance)) or (medication adj2 adherenc*) or ((treatment or vaccin*) adj2 refus*)).ti,ab,kf. | 1 |
| 15 | (patient adj2 (complian* or dropout* or acceptance or participant* or satisfact* or preferenc*)).ti,ab,kf. | 1224 |
| 16 | or/14-15 | 1784 |
| 17 | ((patient* or consumer* or health) adj2 (information or literacy or promotion or education)).ti,ab,kf. | 2988 |
| 18 | 1 and 12 and 16 and 17 | 1 |
| 19 | 13 or 18 | 2 |

**Ovid MEDLINE(R) In-Process & In-Data-Review Citations**

| **#** | **Searches** | **Results** |
| --- | --- | --- |
| 1 | (african adj2 american*).ti,ab,kf. | 1022 |
| 2 | (cultural* adj2 (divers* or character*)).ti,ab,kf. | 136 |
| 3 | (cultural* adj2 adapted).ti,ab,kf. | 73 |
| 4 | (cultural* adj2 competen*).ti,ab,kf. | 175 |
| 5 | (cultural* adj2 tailor*).ti,ab,kf. | 63 |
| 6 | or/2-5 | 429 |
| 7 | (self adj2 (care or monitoring or administration or medication)).ti,ab,kf. | 8972 |
| 8 | ((cerebro* or cerebral* or myocardial or heart or cardiac or Cardiovascular or vascular) adj2 (Disease* or disorder*)).ti,ab,kf. | 14727 |
| 9 | (hypertensi* or infarct or infarction or stroke or strokes).ti,ab,kf. | 14235 |
| 10 | ((glucose adj2 metabolism adj2 (disorder* or disease*)) or (diabetes or diabetic or glycosuria or hyperglycemi* or hyperinsulini* or hypoglycemi*)).ti,ab,kf. | 14165 |
| 11 | (aftercare or rehab*).ti,ab,kf. | 3106 |
| 12 | or/7-11 | 35379 |
| 13 | 1 and 6 and 12 | 4 |
| 14 | ((attitude* adj2 (health or knowledge or practice or compliance)) or (medication adj2 adherenc*) or ((treatment or vaccin*) adj2 refus*)).ti,ab,kf. | 1125 |
| 15 | (patient adj2 (complian* or dropout* or acceptance or participant* or satisfact* or preferenc*)).ti,ab,kf. | 1299 |
| 16 | or/14-15 | 2405 |
| 17 | ((patient* or consumer* or health) adj2 (information or literacy or promotion or education)).ti,ab,kf. | 3376 |
| 18 | 1 and 12 and 16 and 17 | 1 |
| 19 | 15 or 20 | 5 |

**Ovid Embase Classic+Embase**

| **#** | **Searches** | **Results** |
| --- | --- | --- |
| 1 | ancestry group/ or black person/ or african american/ | 127017 |
| 2 | (african adj2 american*).ti,ab,kw. | 93570 |
| 3 | or/1-2 | 148983 |
| 4 | cultural competence/ or cultural diversity/ | 9266 |
| 5 | (cultural* adj2 (competenc* or diversity)).ti,ab,kw. | 6619 |
| 6 | transcultural care/ or cultural nursing/ or indigenous health care/ | 6005 |
| 7 | cultural bias/ or cultural sensitivity/ | 1547 |
| 8 | (cultural* adj2 adapted).ti,ab,kw. | 2469 |
| 9 | (cultural* adj2 tailor*).ti,ab,kw. | 1932 |
| 10 | or/4-9 | 22803 |
| 11 | exp cardiovascular disease/ | 4753345 |
| 12 | ((cerebro* or cerebral* or myocardial or heart or cardiac or Cardiovascular or vascular) adj2 (Disease* or acciden* or disorder*)).ti,ab,kw. | 695396 |
| 13 | (hypertensi* or infarct or infarction or stroke or strokes).ti,ab,kw. | 1448368 |
| 14 | aftercare/ or follow up/ or exp rehabilitation/ | 2192148 |
| 15 | exp diabetes mellitus/ | 1099515 |
| 16 | ((glucose adj2 metabolism adj2 (disorder* or disease*)) or (diabetes or diabetic or glycosuria or hyperglycemi* or hyperinsulini* or hypoglycemi*)).ti,ab,kw. | 1115336 |
| 17 | or/11-16 | 7377104 |
| 18 | 3 and 10 and 17 | 377 |
| 19 | diabetes education/ or health literacy/ or nutrition education/ or patient education/ or health behavior/ or attitude to health/ or health belief/ or health belief model/ or risk reduction/ | 432364 |
| 20 | attitude/ or attitude to health/ or attitude to illness/ or attitude to life/ or exp patient attitude/ | 622021 |
| 21 | patient attitude/ or patient compliance/ or patient attitude/ or dietary compliance/ or medication compliance/ | 234693 |
| 22 | patient attitude/ or patient attendance/ or patient dropout/ or patient participation/ or patient preference/ or patient satisfaction/ or refusal to participate/ or treatment interruption/ or exp treatment refusal/ or vaccine hesitancy/ | 288367 |
| 23 | ((patient* or consumer* or health or nurtition) adj2 (information or literacy or promotion or education)).ti,ab,kw. | 226628 |
| 24 | ((attitude* adj2 (health or to change or attitude to health or cultural bias or cultural sensitiv* or knowledge or practice or compliance)) or (medication adj2 adherenc*) or ((treatment or vaccin*) adj2 refus*)).ti,ab,kw. | 72632 |
| 25 | or/19-24 | 845102 |
| 26 | 3 and 17 and 25 | 1542 |
| 27 | 18 or 26 | 1852 |
| 28 | remove duplicates from 29 | 1832 |

**EBSCOHost CINAHL**

| **#** | **Query** | **Results** |
| --- | --- | --- |
| S1 | MH "Blacks" | 4 |
| S2 | TX (African N2 American*) | 70,581 |
| S3 | S1 OR S2 | 70,584 |
| S4 | (MH "Cultural Competence") OR (MH "Cultural Diversity") OR (MH "Transcultural Care") OR "cultural characteristics" | 25,790 |
| S5 | TX ((cultural* N2 adapted) OR (cultural* N2 competen*) OR (cultural* N2 tailor*)) | 25,181 |
| S6 | S4 OR S5 | 39,072 |
| S7 | (MH "Self Care") OR (MH "Blood Glucose Self-Monitoring") OR (MH "Self Administration") OR (MH "Self Medication") | 51,007 |
| S8 | (MH "Cardiovascular Diseases+") OR (MH "Diabetes Mellitus+") | 757,332 |
| S9 | (MH "After Care") OR (MH "Rehabilitation+") | 324,811 |
| S10 | S7 OR S8 OR S9 | 1,085,963 |
| S11 | (MH "Attitude to Health") OR (MH "Health Beliefs") OR (MH "Patient Compliance") OR (MH "Patient Satisfaction") OR (MH "Patient Preference") OR (MH "Treatment Refusal") OR (MH "Attitude to Illness") | 160,808 |
| S12 | (MH "Cultural Bias") OR (MH "Cultural Sensitivity") | 8,253 |
| S13 | S4 OR S5 OR S12 | 44,381 |
| S14 | S3 AND S10 AND S13 | 579 |
| S15 | (MH "Consumer Health Information") OR (MH "Health Literacy") OR (MH "Health Promotion") OR (MH "Weight Reduction Programs") OR (MH "Patient Education") OR (MH "Diabetes Education") | 162,536 |
| S16 | S3 AND S10 AND S11 AND S15 | 202 |
| S17 | S14 OR S16 | 745 |
