# Supplementary material for: Community-Based Culturally Tailored Education Programs for Black Communities with Cardiovascular Disease, Diabetes, Hypertension, and Stroke: Systematic Review Findings
Source: J Racial Ethn Health Disparities. 2022 Dec 12;10(6):2986–3006. doi: 10.1007/s40615-022-01474-5 (PMC10645635; doi:10.1007/s40615-022-01474-5)
Supplement: Supplementary file 2 — Supplementary file2 (DOCX 50 KB) [file 40615_2022_1474_MOESM2_ESM.docx]

Supplementary material 2: PRISMA Flow

**Identification of studies via databases and registers**

3,211 Records identified from:

CINAHL Embase (n = 745)

Classic+Embase (n = 1,832) MEDLINE(R) (n = 627)

Medline Epub Ahead of Print (n = 2)

Medline-in-Process & In-Data Review (n = 5)

486 Records removed *before screening*:

Duplicate records removed on Endnote (n = 483)

Duplicate records removed on Covidence (n = 3)

**Identification**

Records screened on Covidence (n = 2,725)

Records excluded (n = 2,510)

Reports sought for retrieval

(n = 215)

Reports not retrieved

(n = 12)

**Screening**

Reports assessed for eligibility on Covidence (n = 203)

Reports excluded (n = 136):

Wrong study design/literature type (n = 54)

Wrong program type (n = 54)

Wrong population (n = 25)

Not in English (n = 2)

Wrong Year (n = 1)

Records identified by hand-searching (n = 7)

74 Studies included in review:

-Covidence (n = 67)

-Hand-searched records (n = 7)

**Included**

*From:*  Page MJ, McKenzie JE, Bossuyt PM, Boutron I, Hoffmann TC, Mulrow CD, et al. The PRISMA 2020 statement: an updated guideline for reporting systematic reviews. BMJ 2021;372:n71. doi: 10.1136/bmj.n71
