# Supplementary material for: Community-Based Culturally Tailored Education Programs for Black Communities with Cardiovascular Disease, Diabetes, Hypertension, and Stroke: Systematic Review Findings
Source: J Racial Ethn Health Disparities. 2022 Dec 12;10(6):2986–3006. doi: 10.1007/s40615-022-01474-5 (PMC10645635; doi:10.1007/s40615-022-01474-5)
Supplement: Supplementary file 3 — Supplementary file3 (DOCX 35.1 KB) [file 40615_2022_1474_MOESM3_ESM.docx]

| **First Author, Year** | **Inclusion criteria** | **Sex** | **Sample Age (years)** |
| --- | --- | --- | --- |
| ***Cardiovascular Disease*** | | | |
| Brewer et al., 2017 | >18 years of age, attends worship service at a participating African American church | Female = 26 (70.3%) Male = 11 (29.7%) | Mean = 51.7; Range = 24-79 |
| Villablanca et al., 2016 | High-risk women, African American | All Female | Mean = 59; Range = 37-77 |
| ***Diabetes*** | | | |
| Abbot et al., 2019 | African American, >22 years of age, pre-diabetes or diabetes diagnosis, could understand and speak English | Intervention: Female = 61 (81.3%) Male = 14 (18.7%)  Control: Female = 49 (69.0%) Male = 22 (31.0%) | Intervention: Mean = 61.8 (SD = 12.9)  Control: Mean = 61.6 (SD = 12.8) |
| Anderson et al., 2005 | African American, diabetes diagnosis, lived in Detroit area | Female = 196 (82.0%)  Male = 43 (18.0%) | Mean = 61.0 (SD = 11.4) |
| Anderson-Loftin et al., 2002 | high risk (hemoglobin A1C>8%, cholesterol >200mg/dL, triglycerides >200 mg/dL, low-density lipoprotein -cholesterol >100 mg/dl, weight >25 mg/m^2, summary score on Food Habits Questionnaire >2.5), African American adults with diabetes | Female = 17 (73.0%) Male = 6 (27.0%) | Mean = 51; Range = 25-77 |
| Anderson-Loftin et al., 2005 | African American, type 2 diabetes, 18+years, no mental or physical limitations that would preclude participation in group activities/discussion, >1 diabetes complications (hemoglobin A1C>8%, cholesterol>200 mg/dl, triglycerides >100 mg/dL, weight >25kg/m^2, high fat diet) | Intervention: Female = 38 (78.0%) Male = 11 (22.0%)  Control: Female = 35 (75.0%) Male = 13 (25.0%) | Intervention Group: Mean = 58.9 (SD = 10.1); Range = 40-77  Control Group: Mean = 55.7 (SD = 12.1); Range = 32-86 |
| Austin & Claiborne, 2011 | African American, type 2 diabetes diagnosis, attended 1 of 4 church locations | Unclear | Unclear |
| Beune et al., 2014 | Surinamese or Ghanaian (self-identified), >20 years of age, have received treatment for hypertension, had systolic blood pressure>140 mmHg at last primary care visit | Intervention:  Female = 43 (61%)  Male = 30 (44%)  Control:  Female = 28 (39%)  Male = 38 (56%) | Intervention: Mean 53.3 (SD = 10.2)  Control: Mean 54.6 (SD = 9.5) |
| Bogner & de Vries, 2010 | >50 years of age, Hemoglobin A1C >7 at last primary care office visit or prescribed oral hypoglycemic agent in past year, depression diagnosis or antidepressant prescription | Intervention: Female = 24 (82.8%) Male = 5 (17.2%)  Control: Female = 25 (86.2%) Male = 4 (13.8%) | Total: Mean = 60.2; Range = 50-80  Intervention: Mean = 61.6 (SD = 8.3)  Control: Mean = 58.3 (SD = 6.3) |
| Bray et al., 2005 | type 2 diabetes, had one of the following on day of visit at 2 primary care practices that serve predominantly African Americans: Hemoglobin “A1c, 7.0%, blood pressure 135/85 mm Hg, or physical examination or laboratory evidence of a high risk of end-organ disease including diabetic retinopathy, nephropathy, or neuropathy" | Intervention: Female = 64 (57%) Male = 48 (43%)  Control: Female = 25 (52%) Male = 23 (48%) | Intervention: Mean = 60 (SD = 13)  Control: Mean = 58 (SD = 17) |
| Carter et al., 2011 | Type 2 diabetes, >18 years of age, reside in target area, primary care physician willing to participate in project or willing to be assigned to participating primary care physician, African American, have ability to read >8th grade level | Intervention: - Male = 8 (30.8%) - Female = 18 (69.2%)  Control: - Male = 9 (42.8%) - Female = 12 (57.1%) | Total: Mean = 56; Range = 36-74  Intervention: Mean = 52  Control: Mean = 49 |
| Collins-McNeil et al., 2012 | African American, type 2 diabetes diagnosis, written and verbal comprehension, provide written consent, receive medical clearance from health provider and remain under provider care during study | Male = 2 (16.7%) Female = 10 (83.3%) | Mean = 55.83 (SD = 9.62); Range = 35-68 |
| Crowley et al., 2013 | >18 years of age, Black/African American, >1 primary care visit in past year, type 2 diabetes code used within past 3 years, >1 hemoglobin A1c measurement in past year | Intervention: Female = 126 (69%)  Male = 56 (31%)  Control: Female = 133 (75%) Male = 44 (25%) | Intervention: Mean = 56 (SD = 12)  Control: Mean = 57 (SD = 12) |
| Cummings et al., 2017 | 18-75 years of age, African American women, uncontrolled type 2 diabetes, no end-stage complications/disease | All Female | Normal Distress: Mean = 54.9 (SD = 10.7)  Elevated Distress: Mean = 48.8 (SD = 10.7) |
| D'Eramo-Melkus et al., 2004 | 18-60 years of age, have primary care provider, type 2 diabetes diagnosis, English-speaking, women [black women] | All Female | Mean = 51.7 (SD = 6.88) |
| Funnell et al., 2005 | African American, type 2 diabetes diagnosis (additional criteria unclear) | Unclear | Unclear |
| Garvin et al., 2004 | Racial ethnic groups: African Americans, Cambodians, Chinese, Filipinos, Koreans, Latinos/Hispanics, Samoans, and Vietnamese | African American-Intervention:  Female = 118 (63.0%) Male = 66 (55.9%) | African American-Intervention Mean = 54.9 |
| Gary et al., 2009 | African American, >25 years of age, type 2 diabetes diagnosis | Intervention-Minimal: - Female = 187 (74%) - Male = 66 (26%)  Intervention-Intensive: - Female = 171 (73%) - Male = 64 (27%) | Intervention-Minimal: Mean = 56 (SD = 11)  Intervention-Intensive = 59 (SD = 11) |
| Gore et al., 2012 | African American, >19 years of age | Female = 24 (65%) Male = 13 (35%) | Range = 36-55 |
| Han et al., 2019 | Community-dwelling African Americans; >18 years of age, reside in Baltimore, have uncontrolled diabetes ( hemoglobin A1C>7%) | Female = 12 (63.2%) Male = 7 (36.8%) | Mean = 54 |
| Hassaballa et al., 2021 | High-risk (higher risk of developing a serious diabetes-related complication within 12-months, and were more likely to visit the emergency department and be admitted to the hospital due to their diabetes complications) as determined by a risk algorithm, African American women, >40 years of age | All Female | <40 |
| Hendricks & Hendricks, 2000 | African American, men, type 2 diabetes | All Male | Group 1 (1-month follow-up): Mean = 58.9 (SD = 10.5)  Group 2 (3-month follow-up): Mean = 57.4 (SD = 13) |
| Keyserling et al., 2000 | African American, >40 years of age, female, type 2 diabetes diagnosis at age 20+, no history ketoacidosis | All Female | Mean = 59.2 |
| Keyserling et al., 2002 | African American, >40 years, type 2 diabetes, no history of ketoacidosis | All Female | Group A: Mean = 58.5  Group B: Mean = 59.8  Group C: Mean = 59.2 |
| Lachance et al., 2018 | >18 years of age, African American women, diagnosed with or at risk of diabetes (determined using the American Diabetes Association), reside in Michigan | All Female | Mean = 59.5 |
| Lutes et al., 2009 | rural, 19-75 years old, African American women, self-referred or provider-referred, type 2 diabetes diagnosis and hemoglobin A1c >7.0%, ability to communicate in English, could provide consent | All Female | Mean = 53 (SD = 10.2) |
| Leeman et al., 2008 | African American women, 50-85 years of age, type 2 diabetes | All Female | Range = 50-85 |
| Lynch et al., 2019 | African American, uncontrolled type 2 diabetes (hemoglobin A1c ≥ 7.0%), ≥ 18 years of age, attended a specific primary care clinic in last year, and ability to attend group sessions during specific hours | Female = 148 Male = 63 | Intervention = 55 (SD = 10.3) Control Group = 54.8 (SD = 9.0) |
| Murrocket et al., 2009 | Type 2 diabetes, >18 years, hemoglobin A1C within past 6 months <10%, medical clearance | All female | Treatment Mean 58.5 (12.2)  Control Mean 67.1 (7.9) |
| Okoro, 2020 | Sample from 2 peer support programs (low income, African American, type 2 diabetes, reside in the southeastern region of the US, visited hospital emergency department due to diabetes-related issues which could be prevented by following their diabetes self-care practices, attended peer support >3 times, speak/understand English, no 'mental disability') | Female = 13 (65%) Male = 7 (35%) | Range = 30-82 |
| Peek et al., 2012 | >18 years old, African American, had diabetes, received care from the federally qualiﬁed health center | Female = 88% Male = 12% | Mean = 61 |
| Peǹa-Purcell et al., 2015 | African American, type 2 diabetes diagnosis, willing to participate in study | Intervention: Female = 20 (20.4%) Male = 78 (79.6%)  Control: Female = 3 (21.4%) Male = 11 (78.6%) | Mean = 63.3 (SD = 10.3) |
| Peǹa-Purcell et al., 2019 | African American or Hispanics/Latinos, diagnosed with type 2 diabetes, >18 years of age, complete consent form, willing to participate in study | Female = 181 (77%) Male = 53 (23%) | African American: Mean=66  Hispanics/Lations: Mean= 55 |
| Rovner et al., 2020 | >60 years of age, type 2 diabetes, African American race, hemoglobin A1c of 7.5% or greater, mild cognitive impairment, <80% adherence to oral hypoglycemic medication or insulin | Intervention: Female = 32 (63%) Male = 19 (37%)  Control: Female = 31 (62%) Male = 19 (38%) | Intervention:  Mean = 68.2 (SD = 6.1)  Control: Mean = 68.7 (SD = 6.7) |
| Rovner, & Casten, 2019 | >65 years of age, type 2 diabetes, African American race, mild or moderate nonproliferative diabetic retinopathy, depressive symptoms (Patient Health Questionnaire-9 score ≥ hemoglobin A1C ≥7.0% | Female = 76% | Mean = 68 (SD = 6.1) |
| Ruggiero et al., 2014 | type 2 diabetes for >6 months, taking diabetes medication, Hispanic/Latino or African American, 18+ age, fluent in English or Spanish, recent hemoglobin A1C value ≥6.5%, able to provide informed consent | Intervention:  Female = 91 (68%) Male = 43 (32%)  Control: Female = 92 (70%) Male = 40 (30%) | Intervention: Mean = 53.2 (SD = 11.7)  Control:  Mean = 53.1 (SD = 13.0) |
| Samuel Hodge et al., 2009 | >20 years of age, type 2 diabetes, clinical care provided by a primary care clinician, reside within 50 miles of church for 1 year, have phone access | Intervention:  Female = 75 (64%)  Treatment:  Female = 53 (63%) | Intervention:  Mean = 57 (SD = 0.9)  Treatment:  Mean = 61 (SD = 1.3) |
| Samuel Hodge et al., 2017 | African American, 21–75 years of age, type 2 diabetes; body mass index between 25–47 kg/m2, inclusive; hemoglobin A1c value ≤ 11%; under the care of a health provider, able to perform moderate intensity physical activity, willing to participate with a family partner not diagnosed with diabetes | Participants:  Female = 40 (74%)  Family:  Female 47 (87%) | Participants:  Mean = 54  Family:  Mean = 48 |
| Sharp et al., 2018 | self-identify as African American or Hispanic/Latino, fluent in English or Spanish, have uncontrolled diabetes (HbA1c ≥ 8%) in past year, >21 years of age, received primary care at a specific clinic (UI Health) in past year, have prescription for >1 oral diabetes or hypertension medication | Female: 164 (SD = 67.2)  Male: 80 (SD = 32.8) | Mean = 54.2 (SD = 11.2) |
| Skelly et al., 2005 | Aged 50-85 years, type 2 diabetes, no known cognitive, affective or functional limitations that would preclude participation in the intervention | All Female | Intervention: Mean = 60.5 (SD = 9.0)  Control:  Mean = 63.7 (SD = 10.8) |
| Spencer et al., 2011 | >18 years of age, type 2 diabetes, African American or Latino/Hispanic, lived in target zip code | Intervention: - Female = 54 (75%)  Control: - Female = 62 (67%) | Intervention: Mean = 50  Control: Mean = 55 |
| Steinhardt et al., 2015 | African American, type 2 diabetes, between 30-85 years of age, not participating in another diabetes self-management program | Intervention: Female = 20 (63.6%) Male = 12 (36.4%)  Control: Female = 27 (81.3%) Male = 6 (18.8%) | Intervention: Mean = 50  Control: Mean = 55 |
| Tang et al., 2011 | Diabetes, resident of Ann Arbor/Ypsilanti (Michigan) community, >40 years old, transportation to attend intervention, willing/able to commit 3-4 months to intervention, good baseline communication skills (determined through individual and group interview) | Female = 6 (75%)  Male = 2 (25%) | Mean = 63 years (SD = 7.2)  Range = 48-72 |
| Tang et al., 2005 | 21 or older, type 2 diabetes diagnosis for >1 year, attended a diabetes self-management program in past 3 years, under care of health provider for diabetes management; participants recruited from locations with large African American membership | Female = 50 (81%)  Male = 12 (19%) | Mean = 64.5 (SD = 9.9)  Range = 36-82 |
| Treadwell et al., 2010 | African American men residing in Lorain County, Ohio, at risk or diagnosed with diabetes, poor health may be related to obesity and other health concerns | All Male | Unclear |
| Two Feathers et al., 2005 | African American or Latino, type 2 diabetes diagnosis, >18 years of age, insured or received care from federally qualified health center, ‘mentally able', resided in 1 of the 6 REACH Detroit zip | African American Female = 55 (77.5%) | Mean (African American group) = 60.9 (SD = 13.9) |
| Two Feathers et al., 2007 | African American or Latino, resident of east side or southwest side of Detroit, type 2 diabetes diagnosis | Unclear | Mean = 58.5  Range = 22-90 |
| Utz et al., 2008 | African American, >18 years of age, type 2 diabetes diagnosis, rural country resident, able to provide informed consent | Group DSME: Female = 10 (76.9%) Male = 3 (23.1%)  Individual DSME: Female = 6 (75%) Male = 3 (37.5%) | Group DSME: Mean = 62.4 (SD = 14.7)  Individual DSME: Mean = 56.6 (SD = 14.7) |
| Walker et al., 2010 | African American, >40 years of age, type 2 diabetes | Intervention: Female = 117 (80.7%) Male = 28 (19.3)  Control: Female = 40 (80.0%) Male = 10 (20.0%) | Intervention: Mean = 61.86  Control: Mean = 58.30 |
| Whitney et al., 2017 | African American church member, adult, had diabetes | Focus Group:  Female = 12 (92%)  Pilot Study Group: Female = 14 (78%) | Focus Group: <60 = 7; >60 = 6  Pilot Study Group:  <60 = 7; >60 = 11 |
| Williams et al. 2014 | African American or multiracial, >18 years of age, type 2 diabetes diagnosis, live in rural community, willing to attend 8 sessions | Female = 80% Male = 20% | Mean = 61.9 (SD = 10.8) |
| ***Diabetes and Hypertension*** | | | |
| Lynch et al., 2014 | African American, >18 years of age, prescription medication for type 2 diabetes and hypertension, body mass index 25-45, no medication contraindications to participation | Female = 41  Male = 20 | Mean = 54.1 (SD = 10.0)  Range = 33-77 |
| ***Hypertension*** | | | |
| Bangurah et al., 2017 | Recruited through church that serves predominantly African Americans >55 years of age, diagnosed with or taking oral medications for hypertension, could read and write English at grade 5 level | Female = 12 (75%) Male = 4 (25%) | Range = 55-65 |
| Banks-Wallace, 2007 | African American | Intervention:  Female = 20 (20.4%)  Male = 78 (79.6%)  Control:  Female = 3 (21.4%)  Male = 11 (78.6%) | Mean = 50.3 |
| Bokhour et al., 2016 | Black or African American, documented hypertension >1 uncontrolled blood pressure in past 12 months | Intervention: *N=2 unaccounted Male = 280 (91.5%) Female = 26 (8.6%)  Control: *N=2 unaccounted Male = 283 (91.9%) Female = 25 (8.1%) | Intervention:  - <50 = 29 (9.5%) - 50-65 = 161 (52.4%) - 65+ = 117 (38.1%)  Control:  - <50 = 25 (8.1%) - 50-65 = 161 (51.9%) - 65+ = 124 (40.0%) |
| Boutin-Foster et al., 2016 | African American or Black, hypertension diagnosis, >1 antihypertensive medication and elevated blood pressure at time of study | Intervention: Female = 61 (67.4%) Male = 29 (32.6%)  Control: Female = 61 (70.3%) Male = 26 (29.7%) | Intervention: Mean = 55.45 (SD = 9.26)  Control: Mean = 58.01 (SD = 10.39) |
| Brennan et al., 2010 | >19 years of age, Black or African American, hypertension diagnosis through medical claims in past 18 months, had valid address and phone number, had primary care physician, not participating in another diabetes management program | Intervention: Female = 206  Control: Female = 221 | Intervention Group: Mean = 55.3 (SD = 11.5)  Control Group: Mean = 56.1 (SD = 11.5) |
| Greer et al., 2015 | African American or Black, >18 years of age, primary hypertension diagnosis, systolic blood pressure >140 mm Hg or a diastolic blood pressure >90 mm Hg, ability to read, understand and speak English, prescribed >1 antihypertensive medication | All Female | Mean = 57.98 (SD = 12.37);  Range = 29-86 |
| Gross et al., 2013 | African American clinic patients, >19 years of age, hypertension diagnosis | Female = 28 (80%) Male = 7 (20%) | Mean = 46.4; Range = 29-70 |
| Liang et al., 2015 | African American, 40-75 years of age, uncontrolled hypertension (defined as average blood pressure/2-year period above normal with >1 value >10 mm Hg over normal) | Intervention: Female = 95 Male = 41  Control: Female = 88 Male = 56 | Intervention (compliers) = 62.6 (SD = 8.3)  Intervention (noncompliers) = 60.9 (SD = 8.9)  Control = 61.8 (SD = 10.1) |
| Marseille et al., 2021 | >18 years of age, diagnosis of hypertension, Haitian descent | Female = 25 (59%) Male = 17 (41%) | Mean = 61.95 (SD = 9.75) |
| Meinema et al., 2015 | Specific criteria unclear (participants who completed the CAHE study) | Male = 47.5% | Mean = 53.9 (SD = 9.8) |
| Migneault et al., 2012 | African American, hypertension diagnosis (on active problem list in medical chart), current prescription for >1 antihypertensive medication, 1 primary care visit in past 2 months, two blood pressure readings in past 6 months of >140 mmHg (systolic) and/or >90 mmHg or >130/80 (if diabetic) (diastolic), >35 years old | Intervention:  Female = 111 (65.7%) Male = 58 (34.3%)  Control: Female = 126 (75%) Male = 42 (25%) | Intervention: Mean = 56.3 (SD = 10.6)  Control: Mean = 56.8 (SD = 11.4) |
| Ogedegbe et al., 2012 | African American or Black, fluent in English, hypertension diagnosis, using >1 antihypertensive medication | Intervention: Female = 82%  Control: Female = 77% | Mean = 58 (SD = 12) |
| Resnick et al., 2009 | [African American older adults]: >65 years of age, ability to read/write English, could recall 4 words per the MiniCog, passed the Evaluation to Sign consent, had hypertension or hyperlipidemia and sedentary behavior (<30 minutes/day moderate level physical activity), on antihypertensive or lipid-lowering medications, and managed own medication administration | Female = 64% | Mean = 76.4 (SD = 7.6) |
| Schneider et al., 2005 | African American, residents of West Oakland California or surrounding communities, systolic blood pressure 140-179 mm Hg or diastolic blood pressure 90-109 mm Hg | TM Group:  Female: 25 (46.3%) Male: 29 (53.7%)  PMR Group:  Female: 29 (55.8%) Male: 23 (44.2%)  Control Group:  Female: 25 (56.8%) Male: 19 (43.2%) | TM Group: Mean = 49.3 (SD = 8.9)  PMR Group: Mean = 49.0 (SD = 10.4)  Control Group: Mean = 47.1 (SD 11.0) |
| Schoenthaler et al., 2018 | Black, >18 years of age, diagnosis of hypertension, and uncontrolled blood pressure | Intervention:  Female = 130 (76%) Male = 42 (24%)  Control: Female = 150 (75%) Male = 51 (25) | Intervention: Mean = 63.2 (SD = 11.8)  Control: Mean = 64.1 (SD = 12.1) |
| Scisney-Matlock et al., 2006 | Specific criteria unclear (women from diverse backgrounds) | All Female | Most >63 aged |
| Thomas, & Stoeckel, 2016 | Black men, 65-70 years old, recruited from the cardiology clinic, had hypertension diagnosis, taking different medication for high blood pressure, speak, read and write English | All Male | Range = 65-70 |
| Tully et al., 2015 | Black, uncontrolled hypertension | Intervention: Female = 56%  Control: Female = 55% | Intervention: Mean = 55.8 (SD = 7.1)  Control: Mean = 56.0 (SD = 7.0) |
| Victor et al., 2018 | Non-Hispanic Black men, 35-79 years of age, had systolic blood pressure>140 mm Hg on 2 screening days | All Male | Intervention: Mean = 54.4 (SD = 10.2)  Control: Mean = 546 (SD = 9.5) |
| Victor et al., 2019 | [non-Hispanic Black] Men, 35-79 years of age, regular patrons of participating barbershops, systolic blood pressure >140 mm Hg on 2 screening days | All Male | Intervention: Mean = 54.4 (SD = 10.2)  Control : Mean = 54.5 (SD = 9.4) |
| Webb et al., 2006 | African American women, employed at large metropolitan university | All Female | Mean = 44.8 (SD = 7.6) |
| ***Stroke*** | | | |
| Sajatovic et al., 2018 | <65 years of age, African American, male, had ischemic stroke or transient ischemic attack, planned/recent discharge home, Barthel index score >60, provided informed consent | All Male | Intervention: Mean = 51.3 (SD = 7.0)  Control:  Mean = 53.9 (SD = 7.9) |

Supplementary material 3: Sample characteristics by study
