# Supplementary material for: Community-Based Culturally Tailored Education Programs for Black Communities with Cardiovascular Disease, Diabetes, Hypertension, and Stroke: Systematic Review Findings
Source: J Racial Ethn Health Disparities. 2022 Dec 12;10(6):2986–3006. doi: 10.1007/s40615-022-01474-5 (PMC10645635; doi:10.1007/s40615-022-01474-5)
Supplement: Supplementary file 4 — Supplementary file4 (DOCX 29 KB) [file 40615_2022_1474_MOESM4_ESM.docx]

| **First Author, Year** | **Examples of outcome measures used and reported findings**  *+: significant change at any time during tracking period; NS: non-significant/no-change reported* |
| --- | --- |
| ***Cardiovascular Disease*** | |
| Brewer et al., 2017 | Intervention Feasibility (participation rates, program and speaker evaluations and program scalability): Cardiovascular Health Knowledge Questionnaire: + Cardiovascular Health AHA’s LS7 components-modifiable behavioural factors (physical activity, diet, smoking, adiposity) and biological health factors (BP, cholesterol, glycemic control): NS Outlook on life and health status: NS Psychosocial Measures: NS |
| Villablanca et al., 2016 | Personal CVD risk factors: BMI: NS; waist circumference: +; SBP: NS; DBP: NS; Total and HDL Cholesterol: NS; LDL cholesterol: +; triglycerides: NS; fasting blood sugar: NS) Knowledge-based surveys: knowledge of CVD risk factors: + (increase in knowledge of a low-cholesterol diet); knowledge of symptoms and taking appropriate action by calling 911: +; behaviour Change for heart-healthy lifestyles: +; metabolic and inﬂammatory burden: +) |
| ***Diabetes*** | |
| Abbot et al., 2019 | Diabetes Fatalism Scale: NS/NC Medical Outcomes Study Social Support Survey: NS/NC |
| Anderson et al., 2005 | HbA1C: + Lipids: + Blood pressure: NS/NC Weight: + Diabetes Care Profile (DCP): NS/NC Diabetes Empowerment Scale Short-Form: + Diabetes Attitude Scale-3: NS/NC |
| Anderson-Loftin et al., 2002 | A1C: + Fasting blood glucose test: + Lipid profile: NS/NC Weight: NS/NC Blood pressure: NS/NC Food Habits Questionnaire adaptation: NS/NC Health service use questionnaire: presented as costs Access to care questionnaire: NS/NC Cost of care: NS/NC |
| Anderson-Loftin et al., 2005 | Lipids: NS A1C: NS BMI: + Food Health Questionnaire: + |
| Austin & Claiborne, 2011 |  |
| Bogner & de Vries, 2010 | Medical Outcomes Study Short Form (SF-36): Medication Event Monitoring System (medication monitoring): + A1C: +: NS Center for Epidemiologic Studies Depression Scale (CES-D): + |
| Bray et al., 2005 | Hemoglobin A1c: + Blood pressure: NS Weight: NS |
| Carter et al., 2011 | Blood pressure: NS BMI: + Hemoglobin A1c: +  Brief participant survey of health knowledge, attitudes, behaviours, and practices relative to diabetes and self-perceived physical and mental health status: + |
| Collins-McNeil et al., 2012 | Diabetes Self-Care Practices Measurement Questionnaire: + Self-Appraisal Diabetes Self-Management Scale: NS Center for Epidemiological Studies Depression Scale: NS Spielberger State Anxiety Subscale from the Spielberger State–Trait Anxiety Inventory: NS Perceived Stress Scale: NS Anthropometric measures (total cholesterol; low-density lipoprotein; high-density lipoproteins; triglycerides; hemoglobin A1c; capillary blood glucose: body mass index; systolic blood pressure; diastolic blood pressure; waist circumference; HbA1c: NS |
| Crowley et al., 2013 | Systolic blood pressure: NS HbA1c: NS Low-density lipoprotein cholesterol: NS Morisky Self-reported Medication-Taking Scale: + |
| Cummings et al., 2017 | Systolic BP: NS Weight: NS A1C: NS Diabetes Distress Scale: + Morisky Medication Adherence Scale: +Diabetes Empowerment Scale – Short Form:+ Diabetes Self-Efficacy Scale: "significantly and inversely correlated with improvements in medication adherence, self-care activities, and self-efficacy" |
| D'Eramo-Melkus et al., 2004 | Weight: + A1C: + Fasting blood glucose: + Diabetes Self-Efﬁcacy Outcomes Expectancies: NS Problem Areas in Diabetes: NS BMI: + Diabetes knowledge: NS |
| Funnell et al., 2005 | Unclear |
| Garvin et al., 2004 | Surveys to measure diabetes knowledge, social support level, attitudes, self-efﬁcacy, and health status: + physical activity and dietary behaviours; + diabetes knowledge; + self-efficacy  Focus groups (qualitative) |
| Gary et al., 2009 | Health utilization (emergency room visits and hospitalizations): + HbA1c: NS Lipid profile: favorable for HDL-C and diastolic blood pressure |
| Gore et al., 2012 | Evaluation tool assessing knowledge from program: *descriptive Observations and verbal interactions |
| Han et al., 2019 | Feasibility (overall recruitment level and retention rate); Acceptability (satisfaction survey) HbA1c: effect size 0.27 Fasting glucose: effect size 0.19 Lipids: HDL effect size -0.15 (12 wks) and -0.19 (24 wks); LDL effect size -0.33 (12 wks) and -0.36 (24 wks) Blood pressure: systolic effect size -.14 (12 wks) and .04 (24 wks); diastolic effect size 1.09 (12 wks) and 0.79 (24 wks) Diabetes knowledge test: effect size 0.52 (12 wks) and 0.69 (24 wks) Newest Vital Sign: effect size 0.46 (12 wks) and 0.54 (24 wks) Literacy Assessment in Diabetes: effect size 0.69 (12 wks) and 0.43 (24 wks) Diabetes self-care index custom: effect size 0.29 (12 wks) and 0.15 (24 wks) Stanford Diabetes Self-Efficacy scale: effect size 0.57 (12 wks) and 0.32 (24 wks) Medical Outcomes Study-Social Support Survey: effect size 0.12 (12 wks) and 0.36 (24 wks) Patient Health Questionnaire-9: effect size 0.09 (12 wks) and -0.10 (24 wks) EuroQol:effect size 0.10 (12 wks) and -0.14 (24 wks) EuroQol-5-visual analogue scale: effect size 0.30 (12 wks) and 0.48 (24 wks) |
| Hassaballa et al., 2021 | Emergency department visits: *descriptive (decreased visits by 34%) Hospital admissions: *descriptive (decreased admissions by 40.5%) |
| Hendricks | HbA1c: NS SF-36: NS Diabetes knowledge: NS Healthcare use (hospitalizations, emergency department visits): NR Adherence to medication, dietary, exercise and foot care regime: NS Perception of general health: NS Perceptions of diabetes control: descriptive |
| Keyserling et al., 2000 | Focus groups: qualitative evaluation Effects on diet, physical activity and self-care will be reported in RCT |
| Keyserling et al., 2002 | Intervention acceptability and satisfaction  Physical activity: + |
| Lachance et al., 2018 | Health status: + Diabetes Self-Care Activities Measure -diet: + Diabetes Self-Care Activities Measure -physical activity: NS Health literacy (measured broadly): + Social support questions from Chronic Illness Resources Survey: + |
| Leeman et al., 2008 | Unclear |
| Lynch et al., 2019 | HbA1c: + Blood pressure: NS BMI: NS Nutrition Data System for Research 24-Hour Dietary Recall: NS Alternative Healthy Eating Index 2010: + Accelerometer: NS Nutritional knowledge (adapted Nutrition Knowledge Questionnaire and adultCarbQuiz): + Morisky Medication Adherence Scale: + Patient Health Questionnaire 9: + Support Received subscale from Diabetes Care Profile: NS Social and Personal Factors subscale from Diabetes Care Profile: NS Diabetes Care Profile: NS Newest Vital Sign |
| Lutes et al., 2009 | Glycosylated hemoglobin (HbA1c): NS  Blood pressure: NS  Weight: + |
| Murrocket et al., 2009 | A1C: +  Weight: NS  Body fat: +  Systolic blood pressure: +  Systolic blood pressure: +  Qualitative |
| Okoro, 2020 | Qualitative |
| Peek et al., 2012 | Diabetes self-efﬁcacy 4-item scale: + Diabetes Self-Care Activities Measure: + Decision self-efﬁcacy scale: NS Perception of involvement in care scale: NS HbA1c: + Systolic blood pressure, diastolic blood pressure: NS Cholesterol: + (HDL only)  Weight and BMI: NS Program satisfaction and evaluation-descriptive/qualitative |
| Pena-Purcell et al., 2019 | Kessler-6 questionnaire: + Spoken Knowledge in Low Literacy in Diabetes Scale adapted: NS Diabetes Self-Efficacy Scale: + |
| Rovner et al., 2020 | HbA1c: + (reduction of HbA1c of >0.5% Diabetes Self-Care Inventory-Revised: NS Medication Event Monitoring System: descriptive |
| Rovner, & Casten, 2019 | Diabetes Self-Care Inventory–Revised; Patient Health Questionnaire-9; National Eye Institute Visual Function Questionnaire; Trust single item; Visual acuity and diabetic retinopathy stage A1C: + |
| Ruggiero et al., 2014 | A1C: NS Patient Health Questionnaire, multi-item index of self-confidence |
| Samuel Hodge et al., 2009 | A1C: +  Weight: +  Blood pressure: + (diastolic)  Physical activity actigraph: MI participants modest increase  Fred Hutchinson 12-page Food Frequency Questionnaire: NS  Diabetes Knowledge Scale: +  SF-36 Health Survey: +  Diabetes-related health status: NS  Knowledge and health status instruments: + |
| Samuel Hodge et al., 2017 | Weight: +  BMI: +  Family interaction: +  A1c: +  Self-reported lifestyle and diabetes self-management behaviors: +  Diabetes self-care Composite 7-day score: +  Perceived diabetes negative control: +  Depressive symptom (PHQ8): +  Quality of life (SF21): NS  Blood pressure: NS  Perceptions of diabetes control |
| Sharp et al., 2018 | HbA1c: NS  BMI: NS  Systolic blood pressure: NS  Diastolic blood pressure: +  High and low density lipoprotein-cholesterol: NS  Quality of life (Diabetes Distress Scale): NS  Perceived social support: NS  lipid-lowering medication intensifications: +  Intensification of antihyperglycemic and antihypertensive medications: NS  Diabetes knowledge (Spoken Knowledge in Low Literacy in Diabetes scale)  Depression (Patient Health Questionnaire-2 screen) |
| Skelly et al., 2005 | Diabetes Symptom Distress Scale: NS Diabetes Knowledge Test but too difficult and replaced with New Leaf Diabetes Knowledge Instrument: + HbA1c: NS Quality of Life in Diabetes Instrument: + Diabetes self-care practices instrument: + Participant satisfaction interview: qualitative |
| Spencer et al., 2011 | HbA1c: + LDL cholesterol: within group+/between group NS Blood pressure: NS Self-management knowledge questions: + Problem Areas in Diabetes scale: NS Perceived Competence for Diabetes scale: NS CDC’s Behavioral Risk Factor Surveillance System: within-group +/between group NS |
| Steinhardt et al., 2015 | Connor-Davidson Resilience Scale: NS 4 indicators of resilence: NS Brief version of the Coping Orientations to Problems Experienced Scale: NS Program attendance, satisfaction and retention; *descriptive: high attendance, overall satisfaction and High retention rates Positive Meaning Scale: + Positive and Negative Affect Schedule: NS Social and Personal Factors Scale of the Diabetes Care Profile: NS Perceived Stress Scale: NS Center for Epidemiologic Studies Depression Scale: NS Pedometers: NS Self-Care Inventory-Revised: NS Blood pressure: NS Cholesterol: + (HDL) Triglycerides: NS Fasting blood glucose: + A1C: NS BMI: NS |
| Tang et al., 2011 | Diabetes Knowledge Test Diabetes Knowledge Questionnaire Understanding Management Practice Active Listening Observation Scale Self-efficacy 8-item survey adapted from Heisler & Piette Program satisfaction survey Perceived efficacy of training survey *descriptive |
| Tang et al., 2005 | BMI: + Total cholesterol: + High-density lipoprotein: + Low-density lipoprotein: + Diabetes Quality Improvement Project: + A1C: NS Systolic blood pressure: NS Diastolic blood pressure: NS Problem Areas in Diabetes Scale: + |
| Treadwell et al., 2010 | Weight: Glucose levels: *descriptive - BMI: *descriptive- obesity decreased 50% to 46% among participants Weight: *descriptive - successful weight loss Blood pressure:*descriptive - decreased by 23% 20-question knowledge to assess curriculum:*descriptive Physical activity levels: +*descriptive - increased |
| Two Feathers et al., 2005 | A1C: + Survey on knowledge and behaviors related to diet, physical activity, diabetes self-care activities, and Diabetes-specific quality of life: +  Diabetes Self-Care Activities questionnaire: + Problem Areas in Diabetes Scale: NS Weight: + BMI: NS Blood pressure: NS Medications: NS |
| Two Feathers et al., 2007 | Process Evaluation Measures: descriptive Attendance: descriptive Direct observation, focus groups, and brief questionnaires: descriptive |
| Utz et al., 2008 | AADE 7 Self-Care Behaviors” goal form: NS Diabetes Empowerment Scale- Short Form: NS HbA1C: NS Satisfaction with Diabetes Education Program, ‘Taking Care of Sugar: NS |
| Walker et al., 2010 | BMI: NS HbA1c: NS Weight: NS Diabetes Knowledge Questionnaire: + (knowledge about diabetes) Diabetes Self-Efficacy Outcomes Expectancies Questionnaire: Descriptive Problem Areas in Diabetes Survey: Descriptive Exercise Benefits/Barriers Scale: Descriptive Level of Exercise Stages of Change: Descriptive |
| Whitney et al., 2017 | Unclear |
| Williams et al., 2014 | A1C: NS Diabetes knowledge (Spoken Knowledge in Low Literacy patients with Diabetes (SKILLD) questionnaire): + Diabetes self-efficacy (Diabetes Empowerment Scale-Short Form): NS Daily self-management actions, level of exercise: + Attention to foot care: + Diabetes Problem-Solving Skills: NS A1C: NS Blood lipid: NS Waist circumference: NS Blood pressure: NS BMI: + SF-12: NS Stanford Diabetes Health Care Utilization Form |
| Pena-Purcell et al., 2015 | Diabetes Knowledge Questionnaire: + Diabetes Self-Efficacy Scale: + Psychological Distress Scale: + Healthy Days Measure Scale: + A1C: NS |
| ***Hypertension and diabetes*** | |
| Lynch et al., 2014 | Weight: NS A1c: NS Blood pressure: NS Block Food Frequency Questionnaire: + Community Healthy Activities Model Program for Seniors physical activity questionnaire modified for African Americans: + Morisky Medication Taking Adherence Scale: NS Adapted Nutrition Knowledge Questionnaire: + |
| ***Hypertension*** | |
| Bangurah et al., 2017 | Hill-Bone Compliance to High Blood Pressure Therapy Scale: + Paffenbarger Physical Activity Questionnaire: + Sports/Recreational Activity: + |
| Banks-Wallace., 2007 | Pedometers: +* Cross-Cultural Activity Participation Study Physical Activity Questionnaire (CAPSPAQ): +* Walking diaries: +* blood pressures: +* weight: +* **"Due to variability among individual participants across data collection periods and the overall small sample size, the PI, in consultation with the research team statistician, concluded it is more appropriate to discuss changes in terms of trends rather than statistical significance"* |
| Bokhour et al., 2016 | Post-intervention questionnaire measuring: - engagement with content: + - perceived influence on behaviour change intentions: + |
| Boutin-Foster et al., 2016 | Center for Epidemiologic Studies Depression (CES-D) scale: NS Medication Adherence Self Efficacy Scale: NS Perceived Stress Scale (PSS): NS Positive and Negative Affect Scale (PANAS): NS Medical Outcomes Study-Social Support Survey (MOS-SS): NS |
| Brennan et al., 2010 | Frequency of BP Monitoring: + Blood pressure: + Number of antihypertension medication classes: NS Health care utilization (obtained from the health plan’s claims system): NS |
| Beune et al., 2014 | Systolic blood pressure: NS  Diastolic blood pressure: +  Lifestyle recommendation adherence:+  Morisky medication adherence scale: NS  BMI: NS  medication non-adherence rates: +  sodium excretion: NS |
| Greer et al., 2015 | high BP prevention IQ: NS Hill-Bone Compliance High Blood Pressure Therapy: NS National Health Information Survey: NS Systolic and Diastolic blood pressure: + |
| Gross et al., 2013 | Newest Vital Sign literacy Knowledge tool was developed by the researcher increased knowledge and adherence to the antihypertensive treatment regimen "None of the participants returned for additional education or blood pressure assessments" |
| Liang et al., 2015 | Framingham data CHD risk: NS absolute change in systolic blood pressure: + |
| Marseille et al., 2021 | Hill- Bone Compliance to High Blood Pressure Therapy Scale: + Hypertension Knowledge Test: NS systolic and diastolic blood pressure: + |
| Meinema et al., 2015 | Morisky medication adherence scale: NS Morisky scale (3-item scale): NS Brief Illness Perceptions Questionnaire: + Medication Adherence Self-Efficacy Scale: NS Beliefs about Medicines Questionnaire: NS Duke Social Support Scale: NS Consumer Quality Index-Diabetes: NS Quote Migrant: NS |
| Migneault et al., 2012 | systolic blood pressure: NS 7-day physical activity recall: NS accelerometer: + Diet Quality Score: + Morisky Medication Adherence Scale: NS Blood pressure: NS Intervention acceptability |
| Ogedegbe et al., 2012 | electronic pill monitors: + blood pressure: NS |
| Resnick et al., 2009 | Medication Adherence Self-Efficacy Scale: NS Cardiac Medication Adherence Outcome Expectation Scale: NS Self-efficacy for Health Related Diet: NS single-item diet outcome expectations measure: NS Self-efficacy for exercise scale: NS Positive Outcome Expectations for Exercise scale: NS Negative Outcome Expectations for Exercise: NS Yale Physical Activity Survey: NS Block Brief Food Questionnaire: Blood pressure-systolic: + Blood pressure-diastolic: + |
| Schneider et al., 2005 | Systolic blood pressure: + within but NS between groups Diastolic blood pressure: + change in antihypertensive medication: + within TM group compared to PMR group but PMR and HE groups NS  Block Dietary Food Consumption Questionnaire: NS |
| Schoenthaler et al., 2018 | Systolic blood pressure 6 months: + Diastolic blood pressure 6 months: NS Blood pressure 9 months: + both groups but NS between groups |
| Scisney-Matlock et al., 2006 | Survey Form for the Evaluation The Health Promotion Lifestyle Profile: NS (+ influence of age)  Cognitive Representations of the DASH Diet: + |
| Thomas, & Stoeckel, 2016 | Individual interviews: qualitatively reported |
| Tully et al., 2015 | blood pressure: NS between groups but within intervention group + systolic blood pressure: + diastolic blood pressure: NS  body weight: NS Program evaluation: qualitative |
| Victor et al., 2018 | Systolic blood pressure: +  Diastolic blood pressure: +  Use of antihypertensive medications: + |
| Victor et al., 2019 | Systolic BP: + Diastolic pressure: + Blood pressure goal attainment rates: + Number of antihypertensive: + Drugs prescribed: descriptive Adverse drug reactions: none Primary Care Provider visits: + Self-rated health: + Patient engagement (Patient Assessment of Chronic Illness Care) |
| Webb et al., 2006 | Blood Pressure: NS State-Trait Anger Expression Inventory: NS Personal Strain Inventory: NS Personal Resources Questionnaire: + BMI: NS Abdominal adiposity index: NS |
| ***Stroke*** | |
| Sajatovic et al., 2018 | Tablets Routines Questionnaire: NS systolic blood pressure: + diastolic blood pressure: + HbA1c: + HDL cholesterol: + LDL cholesterol: NS International Physical Activity Questionnaire (IPAQ) short form: + Fagerstrom Test for Nicotine Dependence: NS Hospital Anxiety and Depression Scale: NS Fidelity (compliance and qualitative), Acceptability questionnaire BMI, Addiction Severity Index, need for treatment, Diet Habit Survey, triglycerides: |

Supplementary material 4: Outcome measures used within studies
